# Intestinal Radiation Protection and Mitigation by Second-Generation Probiotic *Lactobacillus-reuteri* Engineered to Deliver Interleukin-22

**DOI:** 10.3390/ijms23105616

**Published:** 2022-05-17

**Authors:** Alexis Espinal, Michael W. Epperly, Amitava Mukherjee, Renee Fisher, Donna Shields, Hong Wang, M. Saiful Huq, Diala Fatima Hamade, Anda M. Vlad, Lan Coffman, Ronald Buckanovich, Jian Yu, Brian J. Leibowitz, Jan-Peter van Pijkeren, Ravi B. Patel, Donna Stolz, Simon Watkins, Asim Ejaz, Joel S. Greenberger

**Affiliations:** 1 Department of Radiation Oncology, UPMC Hillman Cancer Center, University of Pittsburgh, Pittsburgh, PA 15232, USA; espinalam@upmc.edu (A.E.); epperlymw@upmc.edu (M.W.E.); amm223@pitt.edu (A.M.); fisherr3@upmc.edu (R.F.); shieldsd+@pitt.edu (D.S.); huqs@upmc.edu (M.S.H.); diala.hamade.94@gmail.com (D.F.H.); patelr20@upmc.edu (R.B.P.); 2 Department of Biostatistics, University of Pittsburgh, Pittsburgh, PA 15232, USA; how8@pitt.edu; 3 Department of Obstetrics and Gynecology and Reproductive Sciences, University of Pittsburgh, Pittsburgh, PA 15232, USA; avlad@mwri.magee.edu; 4 Department of Medicine, University of Pittsburgh, Pittsburgh, PA 15232, USA; lgc14@pitt.edu (L.C.); buckanovichrj@mwpi.magee.edu (R.B.); 5 Department of Pathology, University of Pittsburgh, Pittsburgh, PA 15232, USA; yuj2@upmc.edu (J.Y.); leibowitzb@upmc.edu (B.J.L.); 6 Department of Food Sciences, University of Wisconsin-Madison, Madison, WI 53706, USA; vanpijkeren@wisc.edu; 7 Department of Cell Biology, University of Pittsburgh, Pittsburgh, PA 15232, USA; donna.stolz@pitt.edu (D.S.); swatkins@pitt.edu (S.W.); 8 Department of Plastic and Reconstructive Surgery, University of Pittsburgh, Pittsburgh, PA 15232, USA; ejaza@upmc.edu

**Keywords:** ionizing irradiation, whole abdomen irradiation, radioprotection, radiation mitigation, *Lactobacillus reuteri*-IL-22

## Abstract

(1) Background: The systemic administration of therapeutic agents to the intestine including cytokines, such as Interleukin-22 (IL-22), is compromised by damage to the microvasculature 24 hrs after total body irradiation (TBI). At that time, there is significant death of intestinal microvascular endothelial cells and destruction of the lamina propria, which limits drug delivery through the circulation, thus reducing the capacity of therapeutics to stabilize the numbers of Lgr5+ intestinal crypt stem cells and their progeny, and improve survival. By its direct action on intestinal stem cells and their villus regeneration capacity, IL-22 is both an ionizing irradiation protector and mitigator. (2) Methods: To improve delivery of IL-22 to the irradiated intestine, we gavaged *Lactobacillus-reuteri* as a platform for the second-generation probiotic *Lactobacillus-reuteri*-Interleukin-22 (LR-IL-22). (3) Results: There was effective radiation mitigation by gavage of LR-IL-22 at 24 h after intestinal irradiation. Multiple biomarkers of radiation damage to the intestine, immune system and bone marrow were improved by LR-IL-22 compared to the gavage of control LR or intraperitoneal injection of IL-22 protein. (4) Conclusions: Oral administration of LR-IL-22 is an effective protector and mitigator of intestinal irradiation damage.

## 1. Introduction

Strategies by which to protect the intestine from ionizing irradiation prior to exposure or mitigation of damage by delivery of agents after exposure have focused on known mechanisms of damage including DNA double strand break repair, mitochondrial mechanisms of apoptosis [1,2,3,4,5,6,7,8,9,10] and other irradiation-induced cell death pathways [11,12]. Radiation-induced death pathways such as necroptosis, parthanatos, ferroptosis, pyroptosis, and intermitotic cell death have been implicated in the intestinal irradiation damage response [11,12]. A common pathway of all forms of irradiation damage involves the depletion of antioxidant repositories within cells, tissues, organs, and organ systems. Initial approaches to radiation protection (delivery of an agent prior to irradiation exposure) have included methods by which to preserve or improve antioxidant stores [11,12]. The administration of cellular antioxidants has been shown to ameliorate irradiation damage if elevated levels can be achieved prior to irradiation exposure [1,2,3]. Both the administration of small molecule radiation protectors and the delivery of transgenes for enzymes involved in the antioxidant response to irradiation (such as manganese superoxide dismutase, peroxidase, and catalase [13,14,15,16]), have been shown to be effective radiation protectors.

Mitigation of radiation damage (delivery of agents after irradiation exposure, but prior to the appearance of symptoms and signs of irradiation damage) has been a more challenging task, given that biochemical steps in irradiation damage are initiated within seconds after exposure [17]. Furthermore, ionizing irradiation damage is dependent upon radiation dose, volume of tissue irradiated, dose rate, and the greater susceptibility of specific cell phenotypes to high linear energy transfer (LET) irradiation, such as that found with protons, neutrons, and charged particles [17].

Recent approaches to intestinal radiation protection and mitigation have derived from an understanding of the cellular and tissue responses that are revealed by levels of the products of stress response genes [18]. Prominent in the cellular response to ionizing irradiation is the upregulation of genes for pathways associated with inflammatory cytokines [11,12,18]. Cascades of elevated cytokines have been demonstrated to follow irradiation exposure to cells in culture, as well as tissues, organs, and organ systems [11,12,18]. Inflammatory cytokine responses to irradiation have been linked to the initial upregulation of promoters of transcription, such as NF-kβ, AP-1, SP-1, and Nrf-2 [18]. The cytokines that are induced by activation of radiation inducible promoters include IL-22 [11,12,18].

Interleukin-22 modulates both acute and chronic inflammatory responses that are associated with several stress induction pathways including those which are initiated by ionizing irradiation [11,12]. IL-22 provides tissue protection in response to many causes of inflammation and infection [19]. IL-22 is produced by both circulating and resident intestinal lymphocytes [19]. The lymphocyte-phenotypes are involved in preserving intestinal barrier function and preventing breakdown, which can lead to intestinal microbes entering the blood [19,20,21,22].

In the present studies, we combined a probiotic LR-IL-22 with two other mitigators, baicalein and metformin, to determine if the combination of mitigators will have a superior radiation mitigative effect of irradiation damage to the intestine. We have demonstrated that *Lactobillus reuteri* (LR) transfected with a plasmid containing the mouse IL-22 transgene was able to mitigate radiation damage [19,23]. LR-IL-22 once gavaged into the intestine lyses and releases the IL-22 into the intestine where it can mitigate irradiation damage to the intestine [19,23]. Baicalein is an anti-ferroptosis radiation mitigator which can mitigate radiation alone or in combination with an anti-apoptosis agent JP4-030 or an anti-necroptosis agent necrostatin [12]. Metformin was the other radiation mitigator we used [24,25,26]. Metformin has antioxidant activity which has been shown to protect normal tissue from irradiation while at the same time making tumor cells more sensitive to irradiation [24,25,26].

## 2. Results

### 2.1. Effect of Gavage of LR-IL-22 on the Ionizing Irradiated Intestine

**Figure 1:**
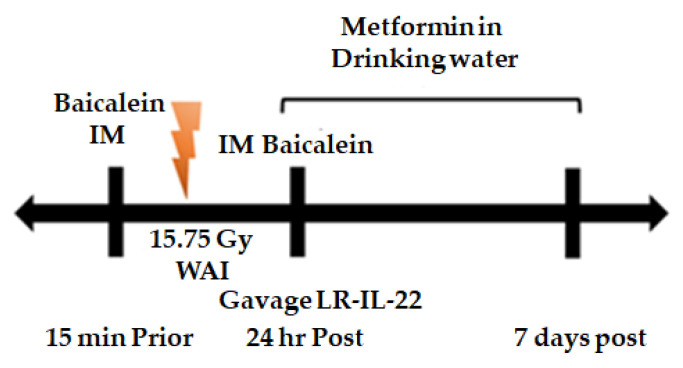
Experimental Paradigm: Small molecule radiation mitigator and probiotic LR-IL-22 administration prior to and following 15.75 Gy WAI. Small molecule mitigators utilized in survival studies include intramuscular Baicalein administered 15 min prior to WAI and 24 h following WAI, Metformin administered orally in drinking water for 7 day following WAI and LR-IL-22 gavaged 24 h following WAI.

**Figure 2:**
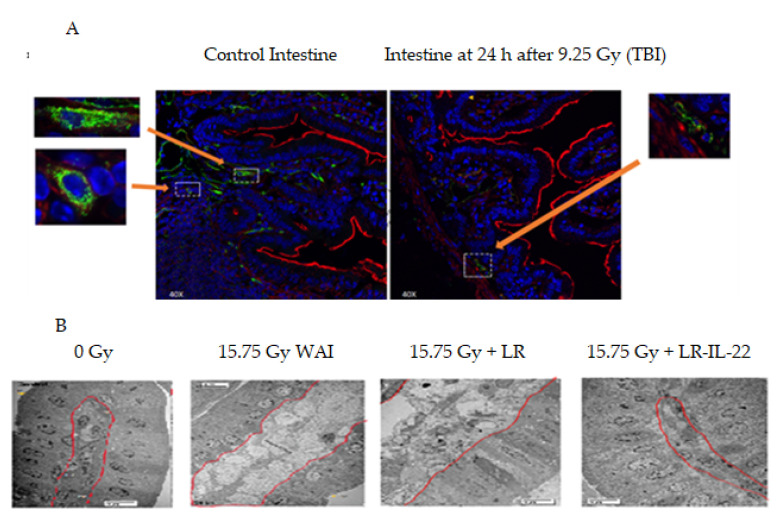
Effect of 9.25 Gy total body irradiation (TBI) or 15.75 Gy whole abdomien irradiation (WAI) observed as destruction of microvascular endothelial cells and cell swelling of the lamina propria including the intestinal multivasculature in the intestinal crypts. (**A**) Control mouse ileum and at 24 h after 9.25 Gy irradiation: Inserts are green = CD31 positive endothelial cells, red = collagen and blue = DAPI (×100). (**B**) Electron micrographs of intestinal villi at 48 h after 15.75 Gy WAI showing cell swelling of lamina propria (×1000). Both 9.25 Gy TBI and 15.75 Gy WAI cause destruction of microvascular endothelial cells and cellular swelling of the lamina propria including the intestinal multivasculature within the crypts. The red line in 2B shows the division of the intestinal villus cells from underlying lamina propria.

We constructed a paradigm for delivery of whole abdomen irradiation (WAI) followed by gavage of LR-IL-22 alone or combined with systemic administration of each of two small molecule radiation mitigators. The paradigm for administration is outlined in (Figure 1). Both total body irradiation (9.25 Gy) and WAI (15.75 Gy) damage intestinal endothelial cells. At 24 h, there was clearly destruction of the endothelial cells in the ileum following 9.25 Gy TBI (Figure 2A). In the irradiated intestine there were fewer CD31 positive endothelial cells and decreased microvasculature compared to the ileum of the control mice which had more CD31 positive cells with an intact microvasculature. Scanning electron microscopy (SEM) following 15.75 Gy WAI revealed that there was damage to the lamina propria (Figure 2B). While the irradiation dose of 15.75 Gy WAI clearly damaged the intestinal microvasculature, the gavage of LR-IL-22 but not control LR ameliorated the damage (Figure 2B). The magnification of areas of the intestinal villus demonstrated integrity of microvascular endothelial cells (CD31+) in those animals receiving LR-IL-22 at 24 h after WAI compared to those receiving radiation alone. SEM of areas of the villus demonstrated damage to not only the lamina propria by 15.75 Gy WAI but also to the other cells in the villus as seen by cellular swelling in the cells, but preservation of the integrity of this anatomic area by gavage of LR-IL-22, but not LR (Figure 2B).

### 2.2. LR-IL-22 Produces Variable Alterations in Intestinal and Plasma Inflammatory Cytokines

**Figure 3:**
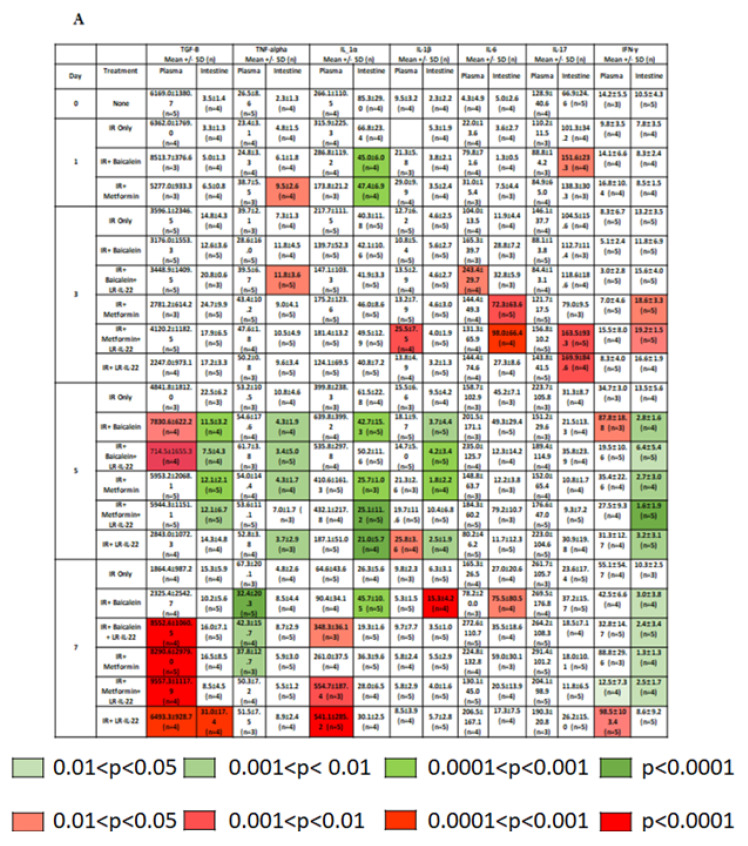

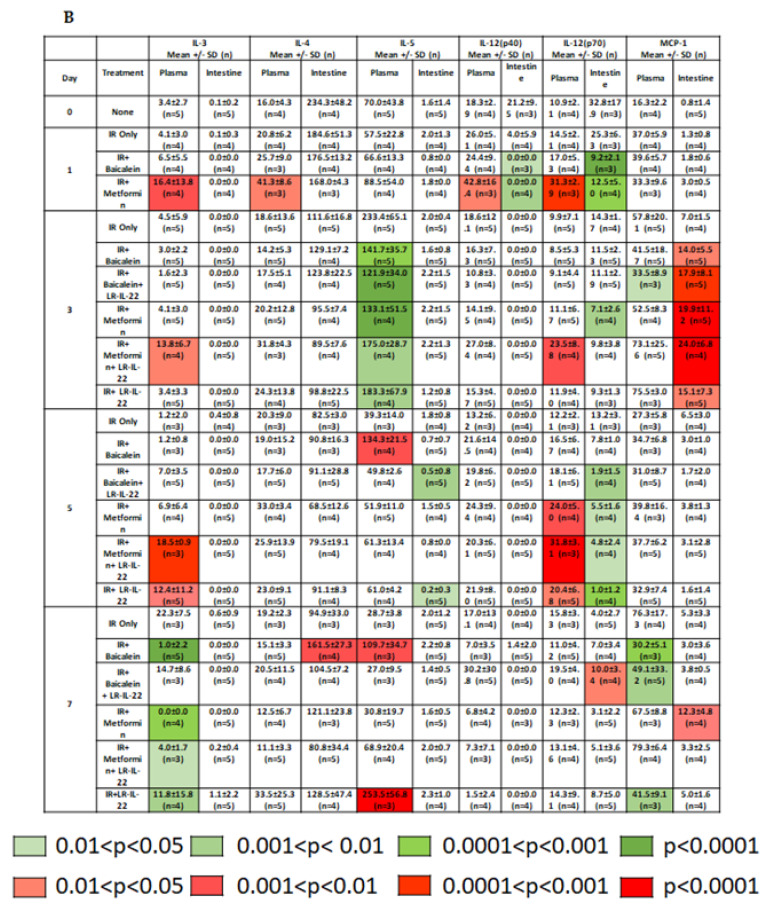

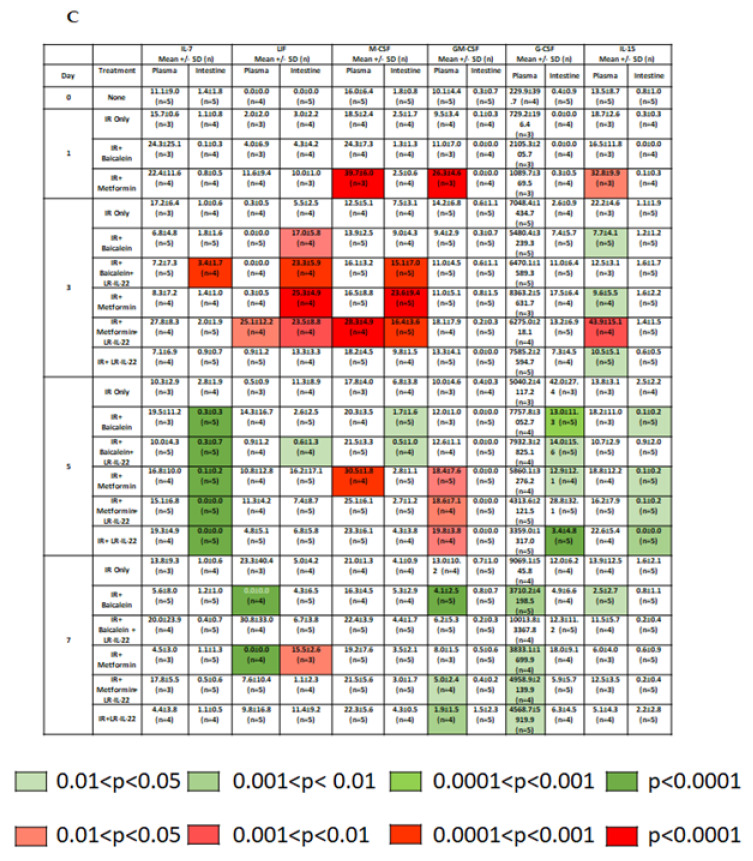

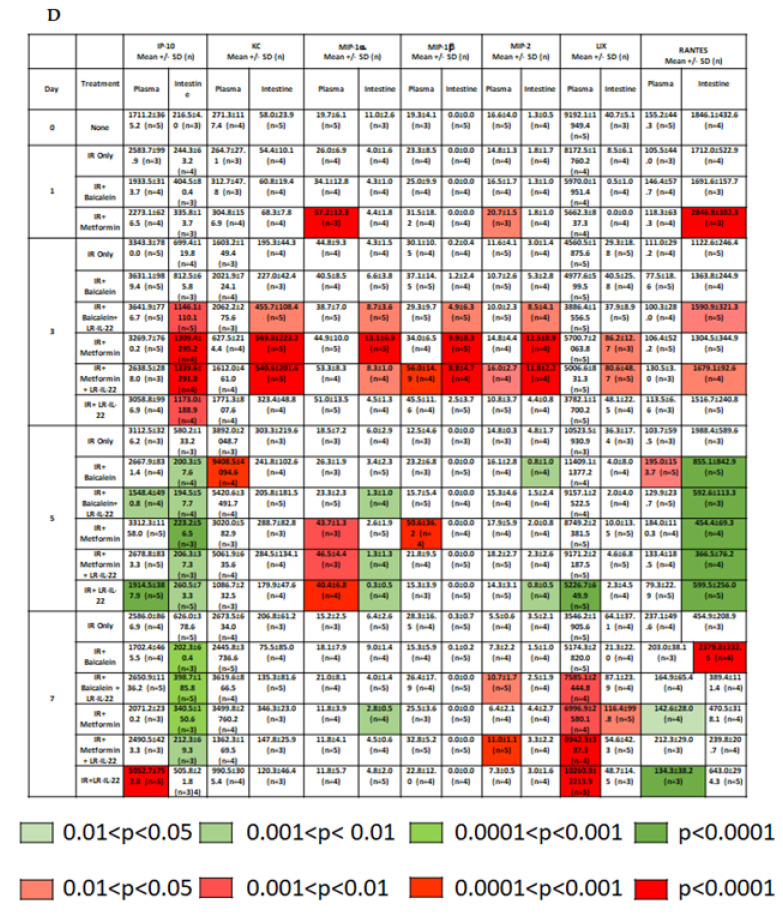

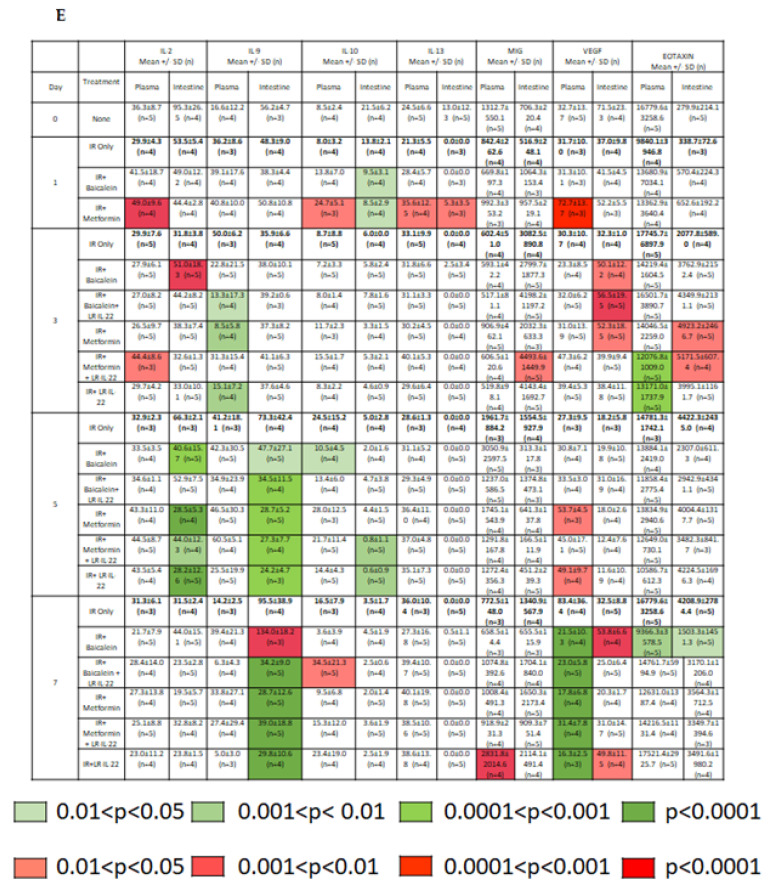
LR-IL-22 modulates irradiation induction of inflammatory cytokine and stress response gene proteins in the intestine and plasma after 15.75 Gy WAI. Six groups of C57 BL/6NTac mice (*n* = 4–5) were irradiated to 15.75 Gy WAI. The groups included: (**1**) irradiation only, (**2**) mice gavaged with 10^9^ copies of LR-IL-22 in 100 μL of saline only; (**3**) metformin only, (**4**) baicalein only, (**5**) LR-IL-22 with metformin, and (**6**) LR-IL-22 with baicalein. Control mice received no irradiation. Mice received LR-IL-22 gavage 24 h following WAI. Mice treated with metformin (60 nM) received drinking water for 7 days following WAI. Mice treated with baicalein (50 mg/kg) received the drug IM 15 min prior to WAI and 24 h after irradiation. Relative levels of expression of each protein ware based in the level detected in the irradiation only group on that same respective day 0, 1, 3, 5, or 7 after irradiation are shown for plasma and intestine panels (**A**–**E**).

We next evaluated the effects of LR-IL-22 oral gavage alone or in combination with intramuscular (I.M.) administration of a systemic radiation mitigator including metformin or baicalein on levels of inflammatory cytokines and stress response genes detectable in the intestine (ileum) or in the plasma. Irradiation produced a statistically significant elevation or depression of the levels of several cytokines in intestinal tissue (ileum) and plasma that participate in inflammation, immunocyte stimulation and chemotaxis, as well as anti-inflammatory cytokines over seven days after 15.75 Gy (Figure 3) (Supplemental Figures S1 and S2). The data in Figure 3 show levels in plasma and intestine (ileum) relative to radiation alone on each of the test days.

Changes in the levels of each of 33 cytokines were observed with both the intestine and plasma in mice treated with gavage of LR-IL-22 alone or combined with systemic delivery of a small molecule radiation mitigator. We tested baicalein, which blocks ferroptosis [12] or the anti-inflammatory drug metformin [24,25,26]. Some of the 33 cytokines were postulated to be pro-inflammatory, such as IL-1alpha (Figure 3A) and were found to be augmented by radiation and lowered by LR-IL-22 with or without small molecular mitigator drugs. We also postulated that protective cytokines, such as LIF and G-CSF (Figure 3C) would be amplified by LR-22 with or without small molecule mitigators, and this result was also observed (Figure 3).

Statistically significant increases in the plasma levels of cytokines in mice treated with LR-IL-22 and either baicalein or metformin relative to levels in mice treated with irradiation alone on each of several days (Day 1, 3, 5, 7 after irradiation) were detected with TGF-B, IL-1a, IL-3, IL-12(p70), LIF, M-CSF, GM-CSF, MIP-1a, MIP-1b, MIP-2, LIX, RANTES, IL-2, and VEGF. Increases in the intestinal levels of cytokines in mice treated with LR-IL-22 and either baicalein or metformin relative to mice treated with irradiation alone on each of several days after irradiation were detected with TNFa, IL-6, IL-17, MCP-1, LIF, M-CSF, IP-10, KC, MIP-1b, VEGF, and Eotaxin (Figure 3 and Supplemental Figures S1 and S2).

Decreases in some cytokine levels were also detected with both the intestine and plasma in mice treated with LR-IL-22 combined with a systemic small molecule mitigator. Decreases in plasma levels of cytokine in mice treated with LR-IL-22 and either bacailein or metformin relative to mice treated with irradiation alone on a given day (Day 1, 3, 5, 7 after irradiation) were observed with TNF-a, IL-3, IL-5, MCP-1, GM-CSF, G-CSF, IP-10, IL-9, and Eotaxin. Decreases in intestinal levels of cytokine in mice treated with LR-IL-22 and either Baicalein or Metformin relative to mice treated with irradiation alone on a given day were observed with TGF-b, TNF-a, IL-1a, IL-1b, IL-5, IL-12 (p70), IL-7, LIF, M-CSF, G-CSF, IL-15, IP-10, MIP-1a, RANTES, IL-2, IL-9, and IL-10 (Figure 3 and Supplemental Figures S1 and S2).

The present results establish that there was significant amelioration of (WAI) irradiation-induced alterations in levels of inflammatory cytokines by gavage of LR-IL-22 and that the changes were detected at several time points following whole abdominal irradiation. At five days following irradiation, there was significant amelioration of irradiation-induced effects on intestinal levels of inflammatory cytokines (TGFb, TNFa, IL-1a) with LR-IL-22 combined with metformin and/or baicalein as opposed to irradiation alone as well as the group treated with irradiation plus LR-IL-22 only. Three days following irradiation, there was restoration of levels of intestinal anti-inflammatory cytokine IL-6 when LR-IL-22 was combined with Metformin and/or Baicalein compared to irradiation alone and compared with mice treated with irradiation, then LR-IL-22 alone. The levels of other anti-inflammatory cytokines including IL-4 and IL-13 did not show a statistically significant elevation or decrease in mice treated with Baicalein or Metformin combined with LR-IL-22 after irradiation.

The present results establish that there was variability in the levels of cytokines at each of several time points following 15.75 Gy (WAI) irradiation. Treatment with each of two systemic mitigators conferred some responses that were measured as changes in radiation-induced cytokine levels. There was also a lower, but detectable effect on cytokine levels by gavage of the control bacteria LR alone.

### 2.3. LR-IL-22 Gavage Mediated Radioprotection of the Intestine Is Enhanced by Systemic Administration of Small Molecule Radiation Mitigators

Previous studies have demonstrated that there is a positive effect of combining two or three small molecule radiation mitigations that target each of three cell death pathways (apoptosis, necroptosis, and ferroptosis) [11,12]. Prior studies also demonstrated that there is a radioprotective and radiation mitigation effect of gavaged LR-IL-22 in an animal model of survival following single fraction TBI [19] and fractionated whole abdomen irradiation (WAI) [22]. Since irradiation to the intestine causes significant destruction of endothelial cells (Figure 2), we tested whether small molecule mitigators that delivered I.M. would be effective in reaching the intestinal villi. Accordingly, we next evaluated the effect on the survival of mice by adding a small molecule radiation mitigator to the protocol of gavage of LR-IL-22 by adding drugs both before and after WAI.

**Figure 4:**
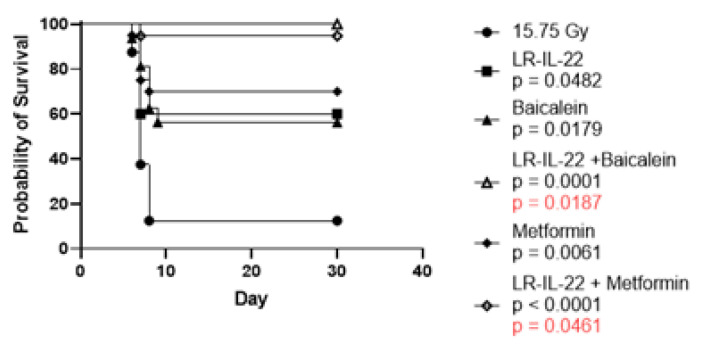
Improved survival of mice administered gavage of LR-IL-22 and intraperitoneal injection of small molecule radiation mitigators in C57BL/6NTac mice administered 15.75 Gy. C57BL/6NTac female mice were irradiated to 15.75 WAI. Subgroups of 20 mice each were treated with baicalein 15 min before and 24 h after WAI. Another subgroup received metformin in the drinking water for 7 days after WAI. Another subgroup was gavaged with LR-IL-22 24 h after irradiation. Other subgroups got LR-IL-22 and either baicalein or metformin as described above. The mice were followed for development of the GI syndrome at which time they were euthanized. GI syndrome is defined as death within 10 days after irradiation of greater that 12 Gy to the abdomen. *p* values values in black are compared to 15.75 Gy WAI only while red *p* values are compared to baicalein only or metformin only.

We evaluated the effect on survival after 15.75 WAI of adding systemic small molecule mitigators to the gavage of LR-IL-22 at 24 h after irradiation. As shown in Figure 4, 90% of mice receiving 15.75 WAI in single fraction died of GI syndrome, which is defined as mice receiving greater than 12 Gy to the abdomen and dying within 10 days of irradiation intestinal damage. These results confirm and extend previous publications on the effect of this dose of WAI [22]. Gavage of LR-IL-22, but not LR or systemic administration of IL-22 protein, improved survival [22], and the results correlated with the preservation of the lamina propria (Figure 2). The addition of a small molecule radiation mitigator to the protocol of WAI followed by LR-IL-22 gavage showed a significant additional improvement in survival in animals that received the anti-ferroptosis drug, baicalein [12], or the antioxidant/anti-inflammatory drug, metformin [26,27] (Figure 4). This agrees with our previous data where we combined either two or three radiation mitigators and increased survival. This also confirms our previous data that baicalein, which is an anti-ferroptosis inhibitor, is a radiation mitigator [12].

### 2.4. LR-IL-22 Effects on Spleen and the Immune System after Irradiation

The physiological effects of ionizing irradiation include the suppression of both humoral and cellular immunity. Radiosensitivity of B and T lymphocytes makes the immune system vulnerable to moderate doses of ionizing irradiation. A mainstay of analysis of radiation exposure has been the measurement of the steepness of the curve demonstrating a drop in peripheral blood lymphocytes [17]. Lymphocyte counts taken at serial 12 h intervals serve as a marker of the relative TBI dose sustained. In analysis of the effectiveness of radiation mitigators, a return of lymphocyte counts has been employed as a sensitive metric for the effectiveness of therapy.

**Figure 5:**
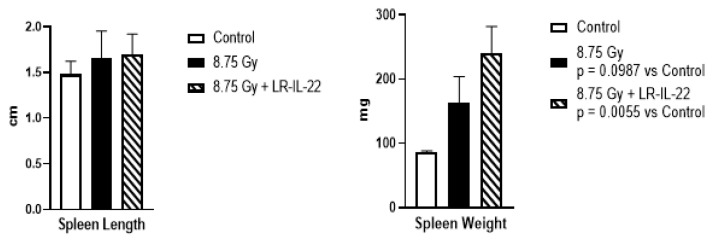
LR-IL-22 stimulates recovery of spleen weight and length at 21 days after total body irradiation (TBI). C57BL/6NTac mice were irradiated to 8.75 Gy TBI. Subgroups of mice were gavaged with LR-IL-22 at 24 h after irradiation. Twenty-one days following irradiation, mice were euthanized and the spleen was removed, weighed, and length measured (*n* = 4 or 5 mice). Results are presented as mean + standard error of the mean.

**Figure 6:**
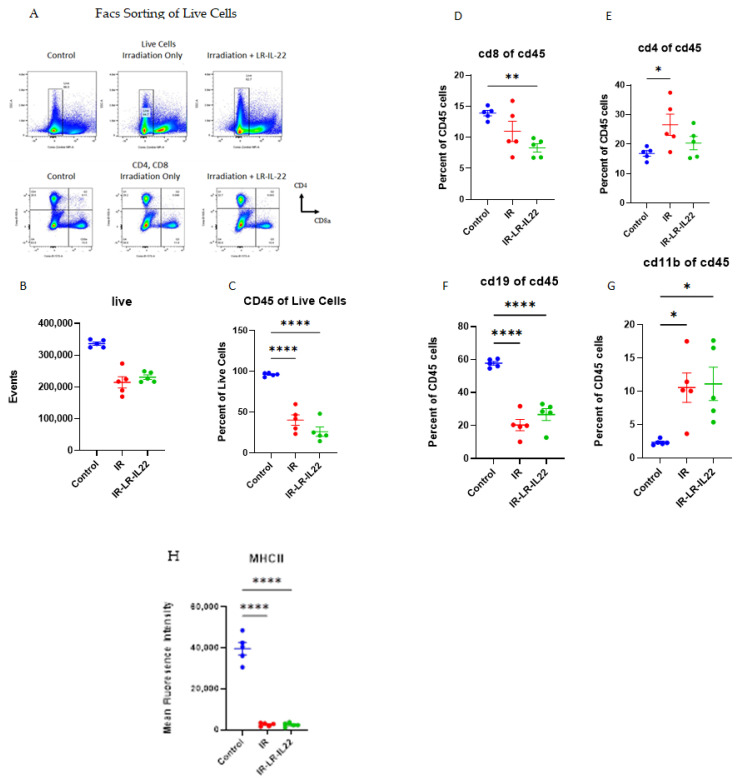
Effect of LR-IL-22 on composition of splenic immunocytes at 21 days after TBI. C57BL/6NTac female mice were irradiated to 8.75 Gy TBI. A subgroup (*n* = 5 mice/subgroup) were gavaged with LR-IL-22 at 24 h after irradiation. Twenty-one days following irradiation the mice were euthanized and the spleen was removed, prepared as single cells suspensions, then stained with antibodies to CD45, CD4, CD8, CD19, CD11b, and MHC11 and analyzed by flow cytometry. (**A**) Flow diagrams, (**B**) Number of live cells in the spleen, (**C**) Percent of live cells that are CD45 positive, (**D**) Percent of CD45 cells that are CD8 positive, (**E**) Percent of CD45 cells that are CD4 positive, (**F**) Percent of CD45 cells that are CD19 positive, (**G**) Percent of CD45 cells that are CD11b, and (**H**) Mean Fluoresence Intensity (MFI) for MHCII expression of CD45 positive cells. Results are plotted as mean ± standard error of the mean. * represents *p* < 0.05, ** represents *p* < 0.01, **** represents *p* < 0.0001 compared to the control mice.

LR-IL-22-stimulated recovery of the spleen was measured by the length and the weight of the spleen at day 21 after 8.75 Gy TBI. This dose of irradiation was chosen since we wanted to measure recovery of the immune cell in the spleen at 21 days after irradiation. If we used higher doses of irradiation the mice will die around day 14 after irradiation due to the development of hematopoietic syndrome. We also quantitated the phenotypes and numbers of cells of each phenotype at day 21 after TBI. Mice receiving gavage of LR-IL-22, but not LR, showed significant enhancement of spleen weight, but less effect on the relative contributions as well as total numbers of immunocytes in each of the categories at day 21. For example, 21 days after TBI, mice treated with LR-IL-22 demonstrated a spleen weight of 240.8 mg compared to a weight of 86.2 mg and 163 mg for control mice and mice treated with irradiation only (Figure 5). Systemic administration of IL-22 protein after irradiation produced some increase in spleen weight, although, not to the level observed with mice that received gavage of LR-IL-22. We evaluated the effects of TBI on the relative percentage and numbers of immunocytes in the spleen. We measured the numbers and weight of relative contributions to the spleen of CD4, CD8 T-cells, dendritic cells, neutrophils, and monocyte/macrophages, as well as, B-cells. Flow analysis of single cell suspensions of spleens removed from mice in each irradiation group are shown in (Figure 6). TBI significantly depleted numbers of immunocytes in all categories compared to unirradiated control mice, with little restorative effect of LR-IL-22 (Figure 6).

The data on flow analysis of the spleen cells at day 21 after irradiation showed that there was a significant decrease in percent of multiple cell phenotypes after irradiation but minimal effect of the LR-IL-22 administration on change in these percentages. Since the spleen weight was increased significantly by LR-IL-22 (Figure 5), the results suggest that the total number of some of these cellular subsets may have, in fact, been increased. In contrast, there was little change in spleen length. The data suggest that the effect of LR-IL-22 was more prominently expressed as an increase in survival, as well as numbers of intestinal stem cells [19] rather than as an increase in numbers of specific immunocytes in the spleen.

**Figure 7:**
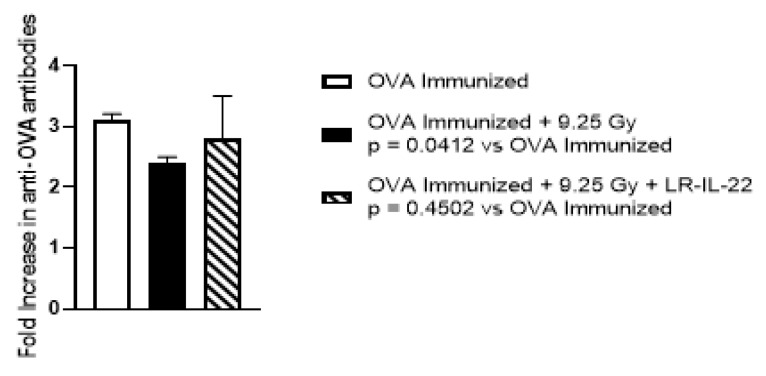
LR-IL-22 stimulates recovery of immunologic function after TBI. C57BL/6NTac mice (*n* = 5 mice per group) were immunized with OVA and the antibody response was determined by ELISA at day 30 and at day 60 following immunization. Following a positive detectable antibody response to OVA on Day 60 after immunization the mice were then irradiated to 9.25 Gy TBI. At 24 after irradiation, a subgroup of mice was gavaged with LR-IL-22. On day 7 after irradiation the mice that had been irradiated were challenged with OVA. At day 11 after irradiation, the mice that had been challenged on day 7 were tested for the antibody response. Irradiation only mice had a significant decreased antibody response compared to OVA immunized mice (*p* = 0.0412) while mice treated with gavage of LR-IL-22 had an antibody response similar to the OVA immunized only mice (*p* = 0.4502). Bar graphs represent mean + standard error of the mean.

To test for radiation-induced changes in immunity, we utilized the chicken ovalbumin mouse immunization model [28] and explored whether memory T-cells and memory B-cells/and/or lymphocyte progenitor cells could be used as a measure of the effectiveness of LR-IL-22 on immune reconstitution. Mice were immunized with OVA [28] and were measured at 30 and 60 days after immunization, when control mice demonstrated significant elevation in antibody to OVA. Experimental mice were subjected to 9.25 Gy TBI (LD70/30 dose). This dose allows us to test the ability of LR-IL-22 on protecting the immunity of the mice under extreme conditions where 70% of the mice will die of hematopoietic syndrome. We sacrificed the mice at day 11 before the mice developed hematopoietic syndrome around day 14, at which time they would have to be euthanized. At 24 h after irradiation, mice were either left untreated or were gavaged with LR-IL-22. Untreated mice, as well as the treated groups, were then followed for a time course and magnitude of recovery of humoral immunity as measured. Immunized mice that were treated with LR-IL-22 demonstrated a significant increase in the level of antibody to OVA compared to non-treated animals (Figure 7). These data support a physiological effect of LR-IL-22 on stimulating the function of the immune system following TBI.

### 2.5. Effect of LR-IL-22 on Bone Marrow

**Figure 8:**
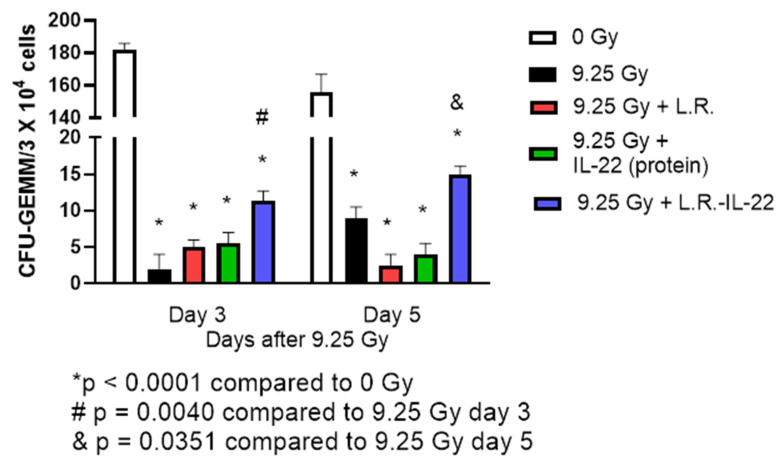
LR-IL-22 gavage at 24 hrs after 9.25 Gy TBI stimulates recovery of bone marrow hematopoietic progenitor cells. Marrow was removed from the femur of C57BL/6NTac mice (*n* = 5) at day 3 or day 5 after 9.25 Gy TBI. Subgroups of mice received irradiation alone or irradiation plus gavage of 10^9^ LR-IL-22 or 10^9^ LR bacteria in 100 μL saline or IL-22 protein at 0.1 mg/kg in 200 μL saline intraperitoneal injection at 24 h after TBI. Marrow was prepared as single cell suspensions and plated at 5 × 10^4^ or 1 × 10^5^ nucleated cells in 0.8% methylcellulose containing medium supplemented with growth factor G-CSF, erythronectin, GM-CSF in McCoys 5A medium plus 15% fetal bovine serum as published (37). Colonies of ≥50 cells were scored at day 14 (*n* = 3 plates per point, *n* = 5 mice per point per group. The * symbol represents the Day 3 irradiation groups to the Day 3 control or the Day 7 irradiation groups to the Day 7 0 Gy group. The # sign is the p value of the Day 3 9.25 Gy + LR-IL-22 compared to the 9.25 Gy group on Day 3. The & symbol represents the p value comparing Day 7 9.25 Gy + IL-22 group to the 9.25 Gy group on Day 7. The data is presented as mean plus standard error of the mean.

In the bone marrow, LR-IL-22 gavage increased the number of hematopoietic progenitor cells forming CFU-GEMM in the femoral bone marrow of TBI mice measured at days one and five after 9.25 Gy TBI (Figure 8). Gavage of LR-IL-22, but not LR, and not *IL-22* protein increased levels of colony forming progenitor cells as an indication of the integrity of the bone marrow microenvironment and hematopoietic stem cells (Figure 8). There was a correlation of the clear increase in survival of irradiated mice following the administration of LR-IL-22 by gavage, with data showing an increase in the numbers of bone marrow colonies forming progenitor cells (Figure 8).

We also evaluated the irradiated mice for the amelioration of the elevation of biomarkers of hepatic and renal damage. Over the seven days after 9.25 Gy TBI, there were no significant changes in levels of hepatic or renal biomarkers including (alkaline phosphatase, ALT, bilirubin, or serum elevation of creatinine). LR-IL-22 gavage did not significantly change the levels of any of these biomarkers. There was no significant change in levels of amylase, a biomarker of pancreas damage.

Collectively, these results demonstrate a protective effect of LR-IL-22 through the preservation of the intestinal barrier, hematopoietic and lymphopoietic organs and adaptive immune function.

## 3. Discussion

### Ionizing Irradiation Results in Dose and Volume Dependent Damage to Multiple Organs and Results in Discrete Syndromes Associated with Irradiation Exposure

Methods by which to ameliorate irradiation damage to the intestine have focused on the systemic administration of radiation mitigators. Some therapeutic cytokines and small molecule radiation mitigators have resulted in improved survival from radiation doses that cause GI Syndrome [19]. One problem with the systemic administration of mitigators has been the radiation damage to the intestine. Irradiation damage to intestinal endothelial cells is detectable at lower radiation doses than that associated with damage to epithelial stem cells or regenerative capacity of the villi [5]. To overcome the potential damaging effects of irradiation on the intestinal microvasculature, which might compromise delivery of systemically administered radiation protectors and mitigators, previous investigators have utilized intraluminal/gavage administration or proteins or small molecules [19]. Oral administration/gavage of small molecules has been limited by potential deleterious degradation-inducing effects of gastric acid on the administered proteins through the stomach into the intestine. The small intestine is known to be significantly radiosensitive compared to the large intestine [1,2,3,4], and, thus, has been studied as the target for delivery of oral/gavage administration of radiation protectors/mitigators. Specific functions of the microbiome are known to be critical for intestinal radiation protection by providing both metabolic functioning and detoxification of deleterious by-products of metabolism, which are important for intestinal radiation protection [27,28,29,30,31,32,33,34,35,36,37,38].

In the present report, we used a bacterial platform *Lactobacillus-reuteri* to deliver a radiation mitigator, IL-22, to the intestine by gavage of a genetically engineered second-generation probiotic, LR-IL-22. IL-22 is a protective cytokine that is part of the IL-10 subset of cytokines and binds to the heterodimer receptor complex composed of IL10-R2 and IL22-R1 [39]. IL-22 is produced by multiple cell types- including Th1 cells such as innate lymphoid cells (ILCs), Th17, and Th22 cells [39]. Dysregulation of IL-22 levels is implicated in multiple clinical diseases, since IL-22 affects the epithelial cells of the intestine, skin, and lung. For example, one study showed the presence of IL-22 producing ILCs in pancreatic cancer, which increased binding to IL-22R in pancreatic tissue and resulted in AKT signaling, which contributed to proliferation, invasion, and migration of cancer cells throughout tumorigenesis. Additional evidence points to increased expression of IL-22 in patients with Crohn’s disease through a Th1- mediated STAT1 and STAT3 activation, further implicating its role in innate immune activation [39].

Systemic administration of IL-22 can rapidly restore bone marrow function and provide rescue from abdominal irradiation-induced intestinal crypt damage, suggesting that a common mechanism may be the replenishment of this cytokine that is naturally produced by intestinal resident lymphocytes, which are the first cells to be eliminated by irradiation. In the present studies, we focused on elucidating the radiation volume dependent, and time dependent, intestinal pathophysiology, and the therapeutic effects of IL-22 administration [19]. Our results reveal that enteric/gavage of LR-IL-22 [19] is superior to systemic administration for delivery of the IL-22 protein in a mouse model of radiation-induced intestinal damage. We also determined whether the improved survival after irradiation by enteric/gavage administration of LR-IL-22 could be further enhanced by systemic administration of other known radiation mitigators.

We previously reported the significant therapeutic effect of the delivery of Interleukin-22 by a second-generation probiotic, Lactobacillus-reuteri, releasing IL-22 [19]. Plasmid mediated delivery of IL-22 in these second-generation probiotics has been associated with significant improvement in survival, preservation of intestinal crypt stem cells, improved intestinal crypt regeneration, and associated improvement in structural integrity [22]. We examined the histopathology of the intestine with representative animals in each group by analysis of several parameters of intestinal barrier function stability, including villus length and integrity of the lamina propria. Animals that received LR-IL-22 demonstrated significant stability of villus length and lamina propria compared to those receiving irradiation alone. Other studies demonstrated that LR-IL-22 gavage preserves levels of intestinal barrier function including the prevention of leakage into the blood of fluorochrome spheres, and preservation of levels of the adhesion molecule proteins occludin and i-CAM [22]. The present histological and ultrastructural data extend these prior results to include the effects of irradiation on the lamina propria of the intestinal villus, which contains the microvascular endothelium, as well as the intestinal immunocytes. There was a low but detectable level of amelioration by gavage of LR alone. These data confirm and extend prior publications demonstrating that Lactobacillus produces small molecule catabolites which ameliorate inflammatory damage to the intestine [19,20,21].

Elucidation of the mechanism(s) of radiation protection and mitigation by LR-IL-22 will require analysis of the several targets of action of this novel therapeutic modality. *Lactobacillus-reuteri*, as well as other strains of *Lactobacillus* have been described as therapeutic probiotics with respect to intestinal health [19]. There is evidence that the relative abundance of *Lactobacillus* in the intestinal microbiome is associated with improved survival after irradiation, as well as other toxin exposures [19,21]. These data relate to the bacterial strain itself, rather than its use as a second-generation probiotic producing a specific cytokine.

Recent attention has focused on the potential importance of anerobic bacterial taxa, including Bacteroides, on intestinal health and recovery from systemic toxins including ionizing irradiation [22,28,29,30]. The use of *E. coli* and particular sub-strains genetically engineered to produce specific small molecules has been tested as a therapeutic probiotic for the treatment of colon cancer [34]. The use of genetically engineered second-generation probiotics to release cytokines have been shown to be effective with both *E. coli* and *L. reuteri* [19]. The potential therapeutic role of *Lactobacillus* itself is supported by our current data showing that control LR has positive therapeutic effects on stimulating the recovery of multiple biomarkers after irradiation exposure, including levels of occludin, i-CAM, and intestinal barrier function, as well as numbers of lgr5+ intestinal crypt stem cells and their regenerative capacity [22].

Our analysis of protein levels in the intestine (ileum) compared to plasma following WAI provided some clues about the mechanism of the therapeutic effect of LR-IL-22. The data revealed several significant changes over the first five days after irradiation exposure. In particular, LR-IL-22 was shown to have a superior effect at reversing/ameliorating these changes compared to LR or the systemic delivery of IL-22. The therapeutic effects of LR-IL-22 may be direct or indirect. IL-22 is known to induce IL-10 and the STING pathway induced type I interferon [4]. In line with these studies, the present data revealed that there were effects of LR-IL-22 on levels of these downstream proteins. These data confirm and extend a prior publication with TBI [12] in mice. Ongoing studies will determine the effects of irradiation and LR-IL-22 gavage on levels of specific gene transcripts in isolated intestinal stem cells and in the progeny of stem cells during the regeneration process.

Our studies provide evidence for the potential therapeutic use of LR-IL-22 protection of the intestine during clinical radiotherapy. In particular, the toxicity of single fraction or fractionated WAI for the treatment of disseminated abdominal ovarian cancer has been a challenge for clinical radiation oncology [22]. This problem has been a particular challenge because ovarian epithelial carcinoma cells are inherently radiosensitive, but the disseminated nature of advanced or recurrent ovarian cancer makes the entire peritoneal surface, as well as the mesentery, at risk for microscopic disease and necessitates the use of target fields that include the entire abdomen including the diaphragm [22]. The primary dose limiting toxicity of WAI has been enteritis. Clinical protocols utilizing WAI in conjunction with combination chemotherapy or immunotherapy have been disappointing and showed no therapeutic advantage to adding a tolerated dose of WAI. Protection of the intestine by gavage of LR-IL-22 between radiation fractions of WAI may afford an opportunity to increase the irradiation dose for WAI in Ovarian cancer and other disseminated abdominal cancers such as peritoneal mesothelioma, abdominal carcinoid tumors, and colon cancer. Studies with animal models of disseminated ovarian cancer utilizing LR-IL-22 gavage to increase the radiation tolerance of the intestine are in progress. The radiation biology of the intestine suggests that enteric/gavage delivery of LR-IL-22 is a good strategy for radioprotection and supports further investigation of the use of second-generation probiotics for therapy of other inflammatory conditions of the intestine.

## 4. Materials and Methods

### 4.1. Animal Care and Usage

C57BL/6NTac (Taconic Biosciences, Renesselar, NY, USA) adult female mice (20–23 gm–6–8 wks old) were maintained with standard laboratory chow and deionized water. Veterinary care by was provided by the Division of Laboratory Animal Research of the University of Pittsburgh. All animal protocols were approved by the University of Pittsburgh Institute of Animal Care and Use Committee of the University of Pittsburgh. The animal usage will show that the combination of LR-IL-22 and baicalein or metformin will protect the mice from irradiation as seen by increased survival following WAI irradiation using Kaplan-Meier plots. To explain how LR-IL-22 protects the intestine, we extracted the intestine and observed changes in histology by immunostaining and scanning microscopy, expression of inflammatory proteins using a Luminex assay, changes in expression of immune cells in the spleen, immune response following ovalbumin (OVA) challenge in OVA immunized mice and bone marrow stem cell activity following 9.25 Gy TBI. In experiments investigating the effects of LR-IL-22 on the immune cells in the spleen, the mice were irradiated to 8.75 Gy to ensure that the mice would survive to day 21 after irradiation and in order not to deplete the immune cells in the spleen so that we would not be able to analyze the effect of LR-IL-22. For the effects of LR-IL-22 on the Ovalbumin challenge and bone marrow we used a dose of 9.25 Gy, which is a LD30/30 dose (30% survival at 30 days after irradiation). This allows us to test immunogenicity and bone marrow stem cell activity under conditions where 70% of the mice will die of hematopoietic syndrome. Power analysis was performed to determine the number of mice to be included in each group for each experiment. For the Kaplan-Meier plot, we will use 20 mice per group. This sample size will provide at least 80% power to detect a 30% difference in 30-day survival between two groups at a one-sided 0.05 significance level. A log rank test will be used to compare two groups to determine significant differences. For the other experiments we will use five mice per group. We assume that the value at each time point has an SD of 15% and that the mean difference between the two groups is 30% and will sacrifice five mice at each time point. This sample size will provide 80% power to detect this difference.

### 4.2. Irradiation

The techniques for total body irradiation (TBI) [11,12] and whole abdomen irradiation (WAI) have been published by us previously. TBI was performed using a JL Shepherd Model 68 Cesium irradiator (JL Shepherd and Associates, San Fernando, CA, USA) at 300 cGy per minute. The mice were placed in a plexiglass mouse irradiation pie plate with five mice placed in the pie plate and including an empty slot between mice. TBI doses of 8.75 Gy and 9.25 Gy were used. In experiments investigating the effects of LR-IL-22 on the immune cells in the spleen, the mice were irradiated to 8.75 Gy to ensure that the mice would survive to day 21 after irradiation and in order to not deplete all of the immune cells in the spleen so that we could not analyze the effect of LR-IL-22. For the effects of LR-IL-22 on the Ovalbumin challenge and bone marrow we used a dose of 9.25 Gy, which is a LD50/30 dose (50% survival at 30 days after irradiation). WAI was performed using a Varian True Beam Irradiator with a 3 cm × 40 cm field. The mice were anesthetized with Nembutal and placed in the irradiation field with the abdomen in the field and the remainder of the mouse out of the field. The mice were irradiated to a single fraction of 15.75 Gy using 600 mKv photons at 600 monitor units per minute at an SSD of 100 cm.

### 4.3. Administration of Interleukin-22

IL-22 (murine) was purchased from Peprotech (Cranbury, NJ, USA) and administered intraperitoneally at 0.1 mg/kg.

### 4.4. Second Generation Probiotic Lactobacillus-reuteri-IL-22 (LR-IL-22)

The methods for construction of LR-IL-22, plasmid-based selection of second-generation probiotics by antibiotic resistance, and standardization of bacterial preparation have been published previously [19]. LR-IL-22 is a second-generation probiotic which contains a plasmid which contains the IL-22 transgene as well as an erythromycin antibiotic resistance gene which allows for selection of the bacteria. LR-IL-22 was grown in De Man, Rogosa and Sharpe (MRS) Broth (Cat#MHA00MRS2, Millipore Sigma, St. Louis, MO, USA) containing 5 ug/mL erythromycin overnight and then diluted with media to an OD of 0.05. Once the bacteria grew to an OD of 0.6, the cells were centrifuged for 5 min at 5000 rpm. The supernatant was poured off and the pellet was resuspended at 10^9^ cells per 200 µL of PBS based upon 1 OD equals 8 × 10^8^ cells.

### 4.5. Preparation and Delivery of Radiation Mitigator Drugs for Survival Analysis

Radiation mitigators included LR-IL-22 at 10^9^ cells in 200 µL of PBS gavaged 24 h after irradiation, baicalein 50 mg/kg (Millipore Sigma, St. Louis, MO, USA) injected intramuscularly 15 min before and 24 h after irradiation and metformin 60 µM in drinking water for seven days, which was initiated immediately following irradiation (Millipore Sigma, St. Louis, MO, USA).

### 4.6. Plasma and Intestinal Protein Level Measurements after WAI Irradiation and Administration of Small Molecule Radiation Mitigators for Luminex Assay

Plasma and Intestinal Protein Levels for Luminex Assay:

A list of 33 proteins assayed and their functions has been published previously [11,12]. Multiple experimental groups were analyzed: 15.75 Gy WAI only, 15.75 Gy WAI and LR-IL-22 24hrs later, 15.75 Gy WAI with Baicalein and LR-IL-22 administration 24hrs later, 15.75 Gy WAI with Metformin administered following WAI and LR-IL-22 administration 24 h following WAI, 15.75 Gy WAI with Baicalein and 15.75 Gy WAI with Metformin administered following WAI. Refer to Figure 1 for timeline of small molecule mitigator and LR-IL-22 administration. A TGF-beta-1 Single Plex Magnetic Bead Kit and 32-Multiplex Mouse Cytokine/Chemokine Magnetic Bead Panel (EMD Millipore, Billerica, MA, USA) were utilized. Protein concentrations for eotaxin, G-CSF, GM-CSF, IFN-γ, IL-1α, IL-1β, IL-2, IL-3, IL-4, IL-5, IL-6, IL-7, IL-9, IL-10, IL-12 (p40), IL-12 (p70), IL-13, IL-15, IL-17, IP-10, KC, LIF, LIX, MCP-1, M-CSF, MIG, MIP-1α, MIP-1β, MIP-2, RANTES, TNF-α, and VEGF were obtained.

### 4.7. Preparation of Standards for Luminex Immunoassay

Mouse cytokine standards were reconstituted in 250 microliters of deionized water, inverted to mix, vortexed, and then transferred to a polypropylene microfuge tube. Serial dilutions were performed using six tubes, by which 50 microliters of standard were inserted into a microfuge tube containing either 200 microliters of assay buffer for 32-multiplex or 150 microliters of assay buffer for TGF-beta-1.50 microliters of this mixture were removed from this microfuge tube and placed into another tube, until all six standards were obtained.

### 4.8. Procedure for 32-Multiplex Luminex Immunoassay and Standardization of Results

Subgroups of female C57BL/6NTac mice were sacrificed by carbon dioxide inhalation according to IACUC approved protocols at serial times after irradiation (1, 3, 5, 7 days post WAI. Blood was obtained in heparinized tubes and was acquired immediately following sacrifice to be processed for the Luminex assay. Intestinal tissue was washed free of blood followed by the addition of 1.0 mL of PBS, homogenized three times for 5 secs on ice using a Brinkman Polytron PT3000 homogenizer (American Laboratory Trading, East Lynn, CT, USA). The homogenate was centrifuged for 5 min at 500× *g* to remove non-lyzed cells and cell fragments with the homogenate isolated and protein determined for use in the Luminex assay.

Prepared standards and controls (25 microliters of each) were added to a specified well followed by 25 microliters of assay buffer to the background sample wells. Twenty-five microliters of (plasma) or 25 microliters 0.1% Tween in PBS was added to background, sample, and control well. A bottle of Pre-mixed beads was vortexed and 25 microliters added to each well. Plates were sealed, covered, and incubated overnight at 2–8 °C. Plates were then placed on a magnet and well contents removed. Plates were washed twice with 200 microliters of wash buffer, 25 microliters of detection antibodies were added to each well and plate was sealed, covered, and incubated for 1 h shaking at room temperature. Twenty-five microliters of streptavidin-phycoerythrin were added to each well and again the plates were sealed, covered, and placed to shake for 30 min at room temperature. After incubation, contents were removed, and plates were washed twice with wash buffer. Lastly, 150 microliters of wash buffer were added to each well, placed to shake for 5 min at room temperature and then read using Luminex 100/200^TM^.

### 4.9. Procedures for TGF-beta-1 Immunoassay-Intestinal Samples

We obtained 25 microliters of each intestinal sample or plasma, and these were placed into individual wells of 96-well plate and treated with 2 microliters of 1.0 N HCl, ensuring that the pH was less than 3.0. The plate was then sealed, covered with aluminum foil, and shaken at room temperature for 15 min. Each sample was then neutralized with 2 microliters of 1.0 NaOH. Please see above for the procedures regarding preparation of a 96-well plate for the 32 Multiplex Luminex Immunoassay.

### 4.10. Procedures of TGF-beta-1 Immunoassay-Plasma Samples

Plasma and intestinal protein were reconstituted in 1.0 mL deionized water and 4.0 mL of Assay buffer was allowed to sit for 10 min at room temperature, at which time 0.1 mL was diluted in 0.5 mL of Assay Buffer (1:30 dilution overall). The procedures regarding preparation of 96-well plate for 32 Multiplex Luminex Immunoassay are as shown above.

### 4.11. Histopathologic Evaluation

Methods for immunohistochemical staining of intestinal ileum have been published previously [1,2,3,4]. Briefly, the intestine samples were fixed in 2% paraformaldehyde for 2 h, transferred to 30% sucrose, and stored at 4 °C. The samples were sectioned, stained with antibodies to CD31 (anti-PECEAM, catalog number 553370, Becton Dickinson Co., Franklin Lakes, NJ, USA), collagen (catalog number ab254113, Abcam, Cambridge, UK), the secondary antibodies Alexa Fluor488 goat anti-mouse IgG and Alexa Fluor594 goat anti-rabbit IgG (A21200 and A11012, respectively, Invitrogen, Waltham, MA, USA), and DAPI (D9542, Sigma-Aldrich, St. Louis, MO, USA) and observed microscopically.

### 4.12. Transmission Electron Microscopy

Intestines were harvested and immersion fixed 2.5% glutaraldehyde, 2% paraformaldehyde in PBS overnight at 4 °C. Following fixation, tissue washed three times in PBS, then post-fixed in aqueous 1% O_S_O_4_, 1% K_3_Fe(CN)_6_ for 1 h. Following three PBS washes, the tissue was dehydrated through a graded series of 30–100% ethanol, 100% propylene oxide, then infiltrated in 1:1 mixture of propylene oxide:Polybed 812 epoxy resin (Polysciences, Warrington, PA, USA) for 1 h. After several changes of 100% resin over 24 h, the tissue was embedded in molds and cured at 37 °C overnight, followed by additional hardening at 65 °C for two more days. Ultrathin (60 nm) cross-sections of intestine were collected on copper grids, stained with 1% uranyl acetate for 10 min, followed by 1% lead citrate for 7 min. Sections were imaged using a JEOL JEM 1400 Flash transmission electron microscope (Peabody, MA, USA) at 80 kV, and imaged with a bottom-mount AMT 2k digital camera (Advanced Microscopy Techniques, Danvers, MA, USA).

### 4.13. Spleen Tissue Collection and Cell Separation

Spleen from female C57BL/6NTac mice was collected under a laminar flow hood under sterile conditions. The weight of the spleen was recorded. Peripheral blood from these same mice was collected via cheek bleeding into heparinized 1.5 mL tubes. Mice spleens were mashed using a sterilized syringe tip. An ACK lysis buffer was added and neutralized by PBS 10 min later. The mixture was then sent through a 70 μm cell strainer, washed twice with PBS, and placed into a 1.5 mL microfuge tube for single cell counting.

### 4.14. Antibodies and Flow Cytometry

The strategy for the flow cytometry gating was to gate the spleen cells for living cells. The living cells were then gated for CD45+ cells which were then analyzed for the presence of CD4, CD8, CD19 or CD11b. Single cell suspensions were resuspended in FACS buffer and treated with Fc Block (BDB553142, Fisher Scientific, Waltham, MA, USA) [37] prior to monoclonal antibody staining. OneComp eBeads Compension Beads (01-111-41, Invitrogen, Waltham, MA, USA) were utilized for antibody compensation control samples. Fluorophore-conjugated antibodies used are as follows: Aqua Live/Dead Fixable Aqua Dead Cell Stain Kit purchased from Biolegend (423106), CD19 (Fisher BD, ID3, BDB749027), CD45 (Biolegend, 30-F11, 103137), CD8a (Biolegend, 53-6.7, 100740), CD11b (Fisher BD, M1/70, BDB563015), CD4 (Fisher BD, GK1.5, BDB563232), TCR γδ (Biolegend, UC7-13D5, 107504), TCRβ (Fisher BD, H57-597, 560705), and MHCIL (Biolegend, M5/114.15.2, 107652). Lymphocytes are defined as live CD45+ gated CD4+ or CD8a+. Myeloid cells were gated on live CD45+ cells. Flow cytometry analysis was performed on a Cytek Aurora using FloJo software. Five thousand events were recorded for compensation controls. 100,000 events were recorded for unstained sample, 40,000 events were recorded for Live-dead, and 500,000 events were recorded for experimental samples [40]. Data are for five mice per group comparing control unirradiated to 8.75 Gy TBI irradiated, or 8.75 Gy irradiated, then gavaged with LR-IL-22 at 24 h. Results are for spleens removed at day 21 after irradiation.

### 4.15. Statistics

For the Luminex data, analyses were done separately for each cytokine and tissue (plasma or intestine) combination. In these analyses, cytokine expression was summarized in each subgroup with mean ± standard deviation (SD) (*n* = 5). At each day of measurement, we compared each treatment group with day 0 data (i.e., non-irradiation control). We also compared the daily data for each group with the irradiation only control on that same day. All comparisons were done based on a one-way ANOVA model, followed by a t-test using ESTIMATE statement in Proc GLM of SAS 9.4 (SAS Institute Inc., Cary, NC, USA). We used the one-way ANOVA test, because the treatments on different days were not identical. In these analyses, a value of *p* < 0.05 was regarded as significant. As these were exploratory analyses, we did not adjust p-values for multiple comparisons.

For other experiments involving multiple groups, such as the experiments in Figure 6 and Figure 7, the one-way ANOVA test was followed by Tukey’s multiple comparison tests. For the mouse survival data in Figure 4, Kaplan-Meier survival curves were plotted for each group. Each treated group was compared to the radiation-only control using the two-sided log-rank test.

## 5. Conclusions

Due to intestinal irradiation damage to the microvasculature, the gavage delivery of the therapeutic radiation mitigator, IL-22, is more effective when administered by gavage (intra-oral) delivery of a genetically engineered second-generation probiotic, LR-IL-22. This new therapeutic should have a wide range of clinical applications in the treatment of many causes of inflammatory damage to the small intestine.

## 6. Patents

Inventor: Xichen Zhang (University of Pittsburgh.); Michael W. Epperly (University of Pittsburgh); Joel S. Greenberger (University of Pittsburgh.) Title: Protection of Small Intestine by Genetically Engineered Probiotics. Our Ref. No.: 05065 Subject: University Ref. No. 05065 Your File: 05065 Pitt Our File: 10504-042PV1 (Lisa C. Pavento, Principal, Meunier Carlin & Curfman LLC).

## Data Availability

Supplemental Data.

## Supplementary Materials

The following supporting information can be downloaded at: https://www.mdpi.com/article/10.3390/ijms23105616/s1.
