# Supplementary material for: Intestinal Radiation Protection and Mitigation by Second-Generation Probiotic Lactobacillus-reuteri Engineered to Deliver Interleukin-22
Source: Int J Mol Sci. 2022 May 17;23(10):5616. doi: 10.3390/ijms23105616 (PMC9145862; doi:10.3390/ijms23105616)
Supplement: Supplementary file 1 [file ijms-23-05616-s001.zip › ijms-1669915-supplementary.pdf]

**Supplemental Figures:** Supplemental Figure S1: Intestine levels of each of 33 cytokines over 7 days after WAI. Supplemental Figure S2: Plasma levels of each of 33 cytokines over 7 days after WAI.

Supplemental Figure S1:

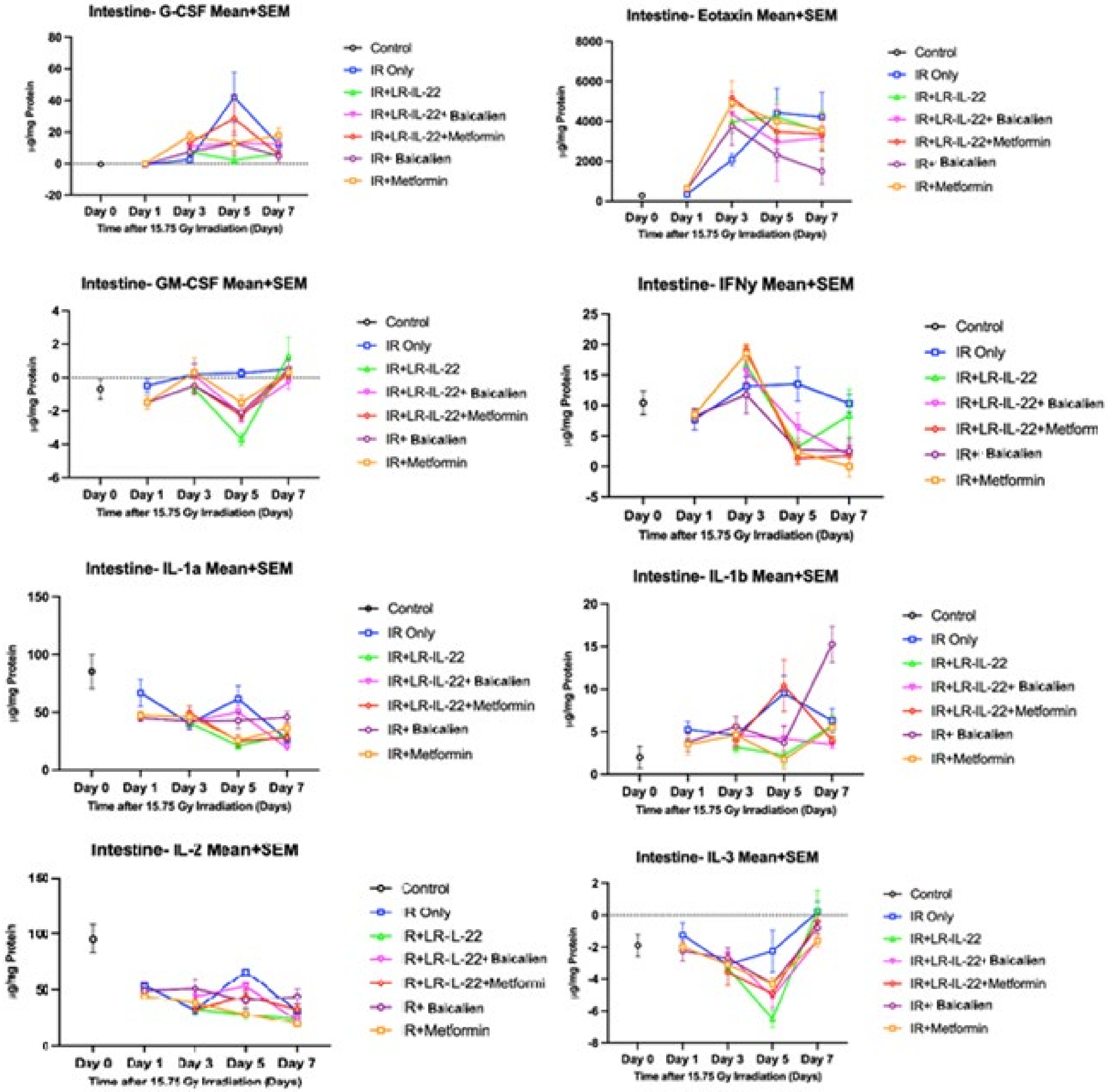

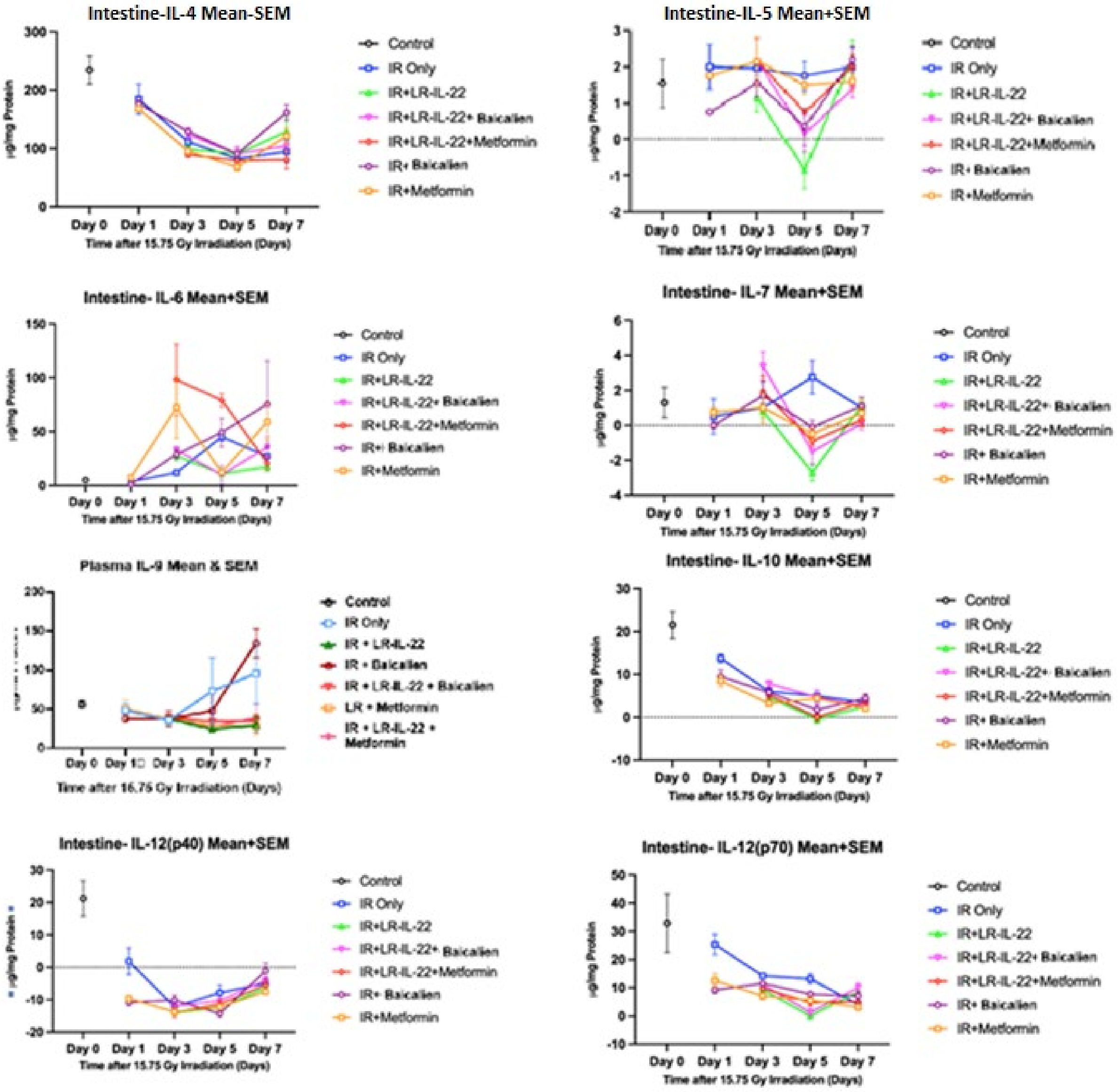

Intestine-IL-4 Mean-SEM
μg/mg Protein
Time after 15.75 Gy Irradiation (Days)
Day 0
Day 1
Day 3
Day 5
Day 7
Control
IR Only
IR+LR-IL-22
IR+LR-IL-22+ Baicalien
IR+LR-IL-22+Metformin
IR+ Baicalien
IR+Metformin
Intestine-IL-5 Mean+SEM
Intestine- IL-6 Mean+SEM
Intestine- IL-7 Mean+SEM
Plasma IL-9 Mean & SEM
IR + LR-IL-22
IR + Baicalien
IR + LR-IL-22 + Baicalien
LR + Metformin
IR + LR-IL-22 + Metformin
Time after 16.75 Gy Irradiation (Days)
Intestine- IL-10 Mean+SEM
Intestine- IL-12(p40) Mean+SEM
Intestine- IL-12(p70) Mean+SEM

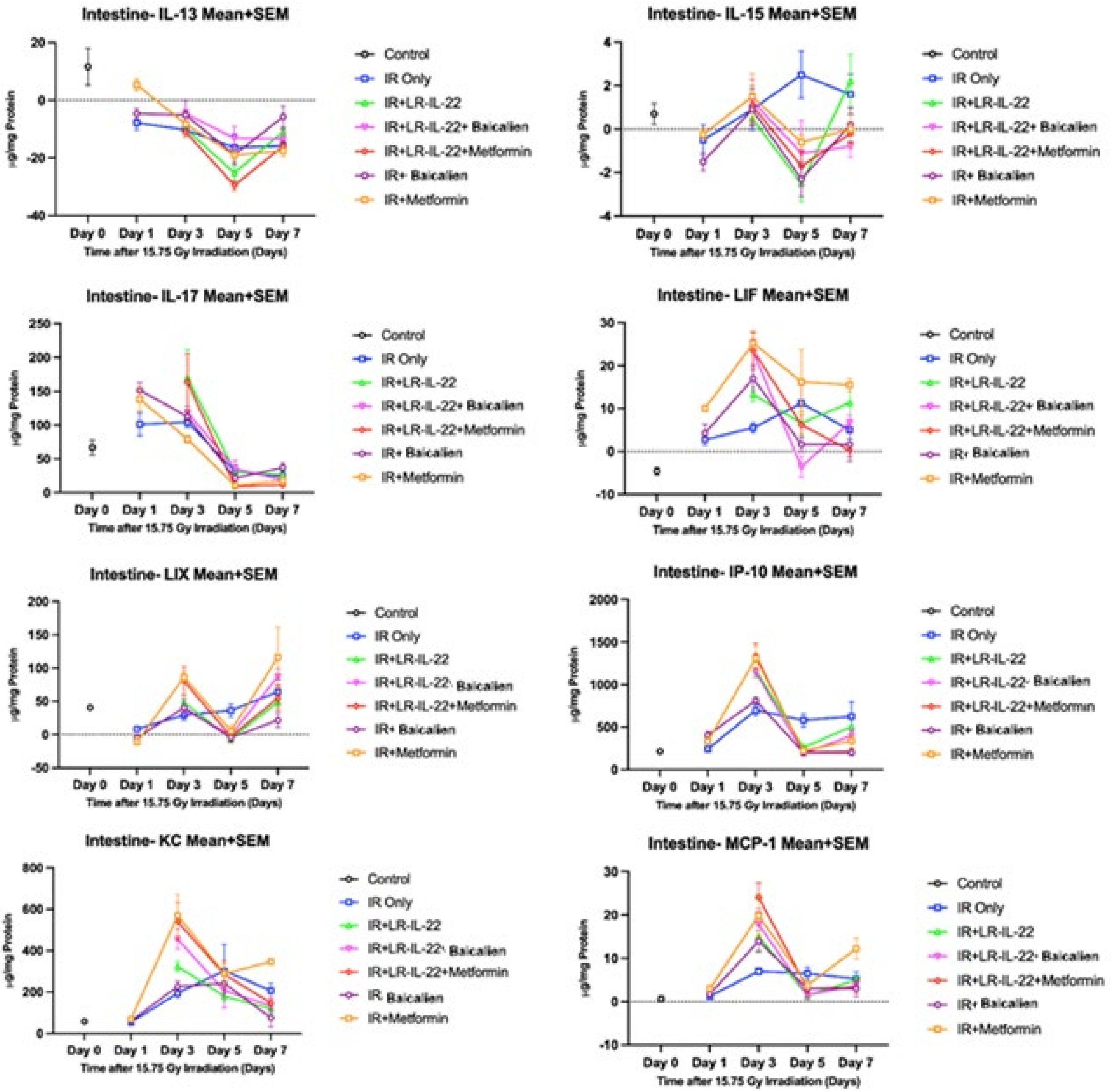

Intestine- IL-13 Mean+SEM
Intestine- IL-15 Mean+SEM
Intestine- IL-17 Mean+SEM
Intestine- LIF Mean+SEM
Intestine- LIX Mean+SEM
Intestine- IP-10 Mean+SEM
Intestine- KC Mean+SEM
Intestine- MCP-1 Mean+SEM
μg/mg Protein
Day 0
Day 1
Day 3
Day 5
Day 7
Time after 15.75 Gy Irradiation (Days)
Control
IR Only
IR+LR-IL-22
IR+LR-IL-22+ Baicalien
IR+LR-IL-22+Metformin
IR+ Baicalien
IR+Metformin

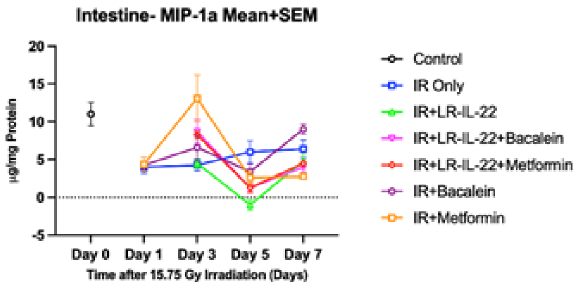
Intestine- MIP-1a Mean+SEM
μg/mg Protein
Day 0
Day 1
Day 3
Day 5
Day 7
Time after 15.75 Gy Irradiation (Days)
Control
IR Only
IR+LR-IL-22
IR+LR-IL-22+Bacalein
IR+LR-IL-22+Metformin
IR+Bacalein
IR+Metformin

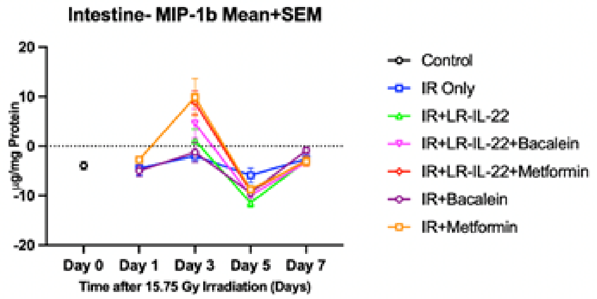
Intestine- MIP-1b Mean+SEM
μg/mg Protein
Day 0
Day 1
Day 3
Day 5
Day 7
Time after 15.75 Gy Irradiation (Days)
Control
IR Only
IR+LR-IL-22
IR+LR-IL-22+Bacalein
IR+LR-IL-22+Metformin
IR+Bacalein
IR+Metformin

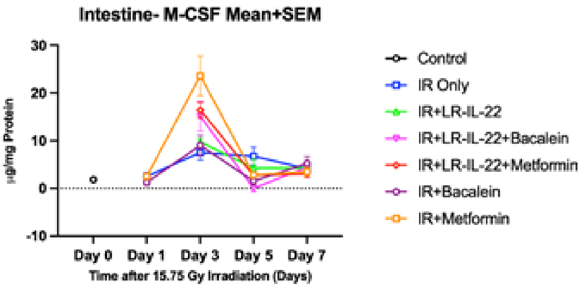
Intestine- M-CSF Mean+SEM
μg/mg Protein
Day 0
Day 1
Day 3
Day 5
Day 7
Time after 15.75 Gy Irradiation (Days)
Control
IR Only
IR+LR-IL-22
IR+LR-IL-22+Bacalein
IR+LR-IL-22+Metformin
IR+Bacalein
IR+Metformin

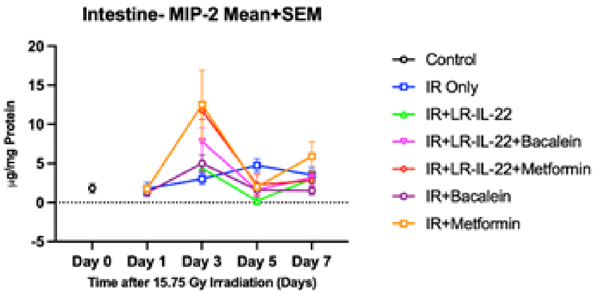
Intestine- MIP-2 Mean+SEM
μg/mg Protein
Day 0
Day 1
Day 3
Day 5
Day 7
Time after 15.75 Gy Irradiation (Days)
Control
IR Only
IR+LR-IL-22
IR+LR-IL-22+Bacalein
IR+LR-IL-22+Metformin
IR+Bacalein
IR+Metformin

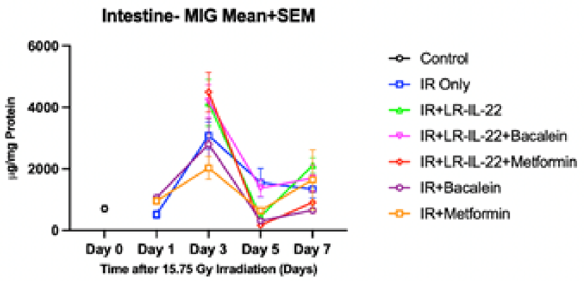
Intestine- MIG Mean+SEM
μg/mg Protein
Day 0
Day 1
Day 3
Day 5
Day 7
Time after 15.75 Gy Irradiation (Days)
Control
IR Only
IR+LR-IL-22
IR+LR-IL-22+Bacalein
IR+LR-IL-22+Metformin
IR+Bacalein
IR+Metformin

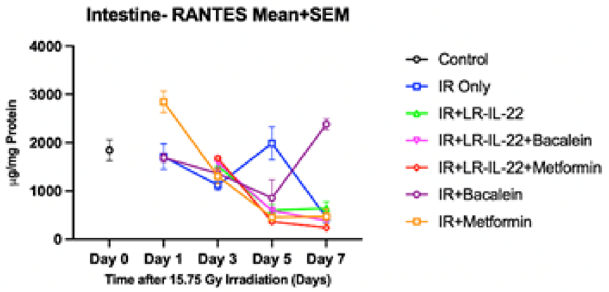
Intestine- RANTES Mean+SEM
μg/mg Protein
Day 0
Day 1
Day 3
Day 5
Day 7
Time after 15.75 Gy Irradiation (Days)
Control
IR Only
IR+LR-IL-22
IR+LR-IL-22+Bacalein
IR+LR-IL-22+Metformin
IR+Bacalein
IR+Metformin

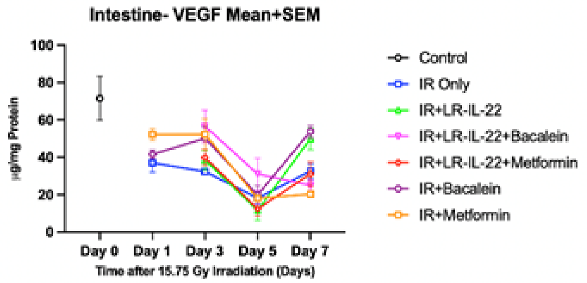
Intestine- VEGF Mean+SEM
μg/mg Protein
Day 0
Day 1
Day 3
Day 5
Day 7
Time after 15.75 Gy Irradiation (Days)
Control
IR Only
IR+LR-IL-22
IR+LR-IL-22+Bacalein
IR+LR-IL-22+Metformin
IR+Bacalein
IR+Metformin

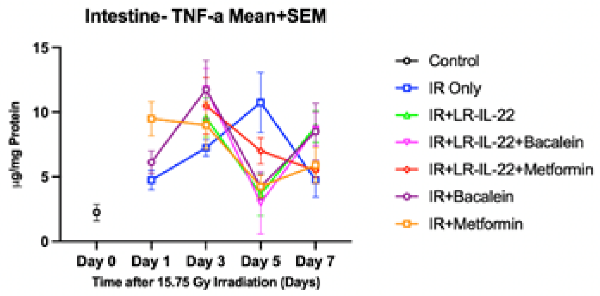
Intestine- TNF-a Mean+SEM
μg/mg Protein
Day 0
Day 1
Day 3
Day 5
Day 7
Time after 15.75 Gy Irradiation (Days)
Control
IR Only
IR+LR-IL-22
IR+LR-IL-22+Bacalein
IR+LR-IL-22+Metformin
IR+Bacalein
IR+Metformin

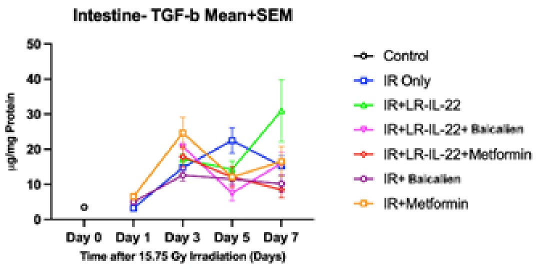
Intestine- TGF-b Mean+SEM
μg/mg Protein
Day 0
Day 1
Day 3
Day 5
Day 7
Time after 15.75 Gy Irradiation (Days)
Control
IR Only
IR+LR-IL-22
IR+LR-IL-22+ Baicalien
IR+LR-IL-22+Metformin
IR+ Baicalien
IR+Metformin

**Supplemental Figure S2:**

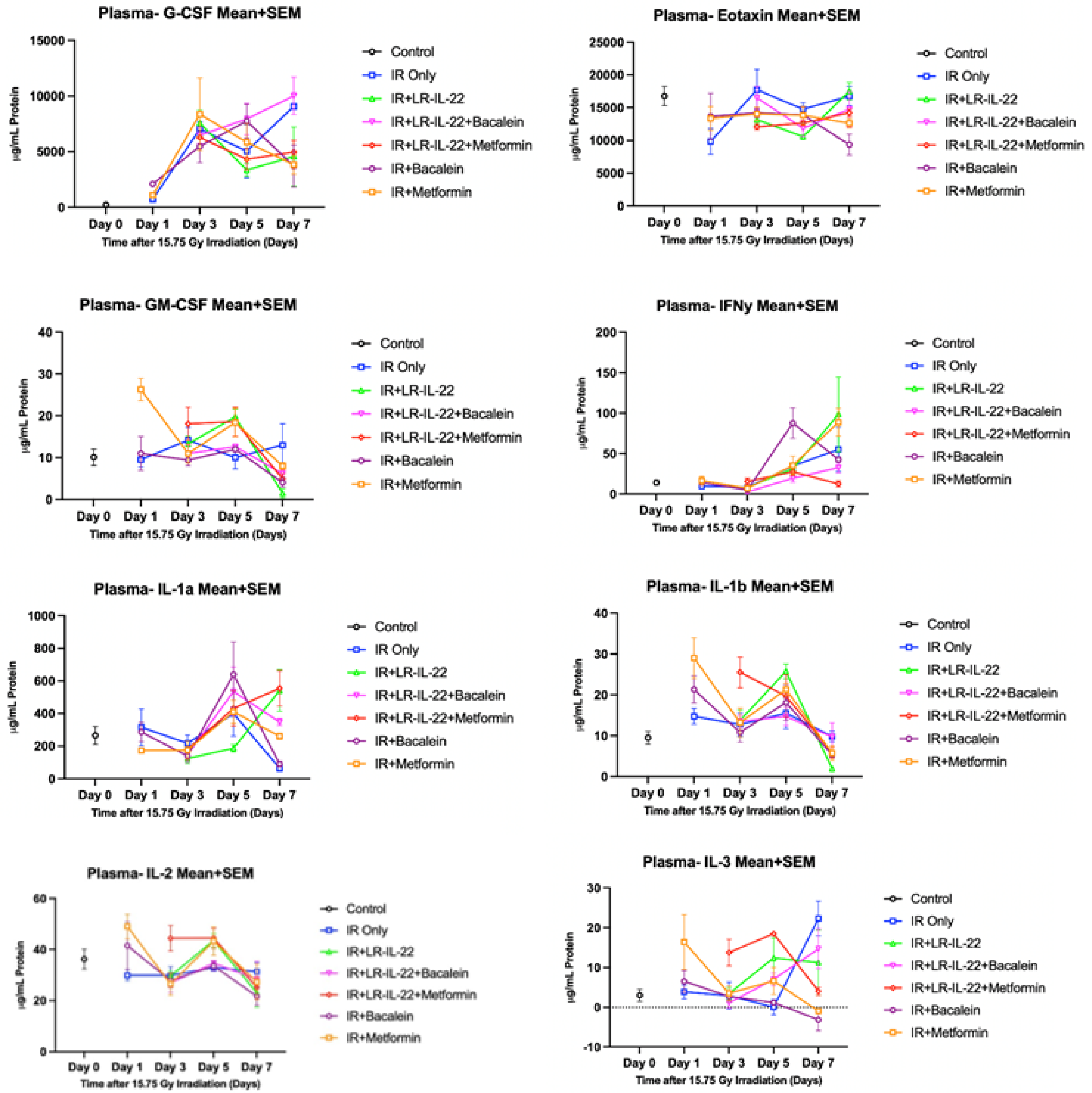

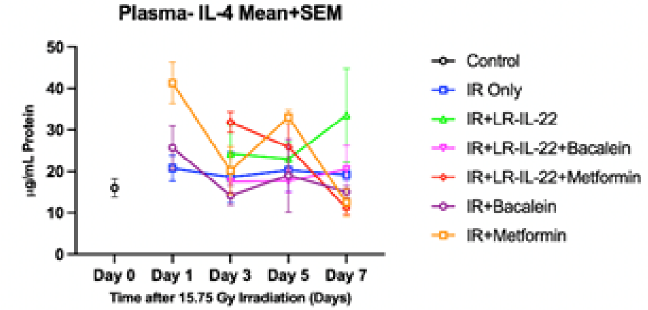
Plasma- IL-4 Mean+SEM
µg/mL Protein
50
40
30
20
10
0
Day 0
Day 1
Day 3
Day 5
Day 7
Time after 15.75 Gy Irradiation (Days)
Control
IR Only
IR+LR-IL-22
IR+LR-IL-22+Bacalein
IR+LR-IL-22+Metformin
IR+Bacalein
IR+Metformin

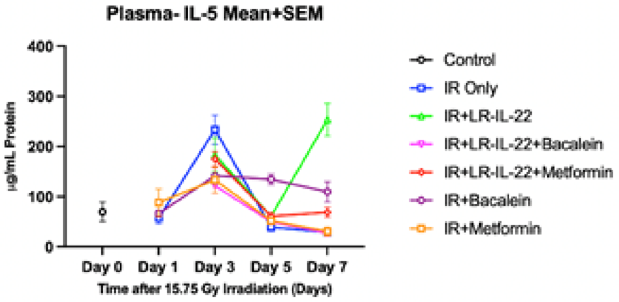
Plasma- IL-5 Mean+SEM
µg/mL Protein
400
300
200
100
0
Day 0
Day 1
Day 3
Day 5
Day 7
Time after 15.75 Gy Irradiation (Days)
Control
IR Only
IR+LR-IL-22
IR+LR-IL-22+Bacalein
IR+LR-IL-22+Metformin
IR+Bacalein
IR+Metformin

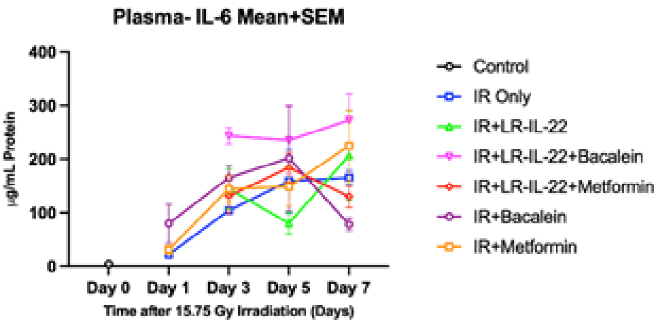
Plasma- IL-6 Mean+SEM
µg/mL Protein
400
300
200
100
0
Day 0
Day 1
Day 3
Day 5
Day 7
Time after 15.75 Gy Irradiation (Days)
Control
IR Only
IR+LR-IL-22
IR+LR-IL-22+Bacalein
IR+LR-IL-22+Metformin
IR+Bacalein
IR+Metformin

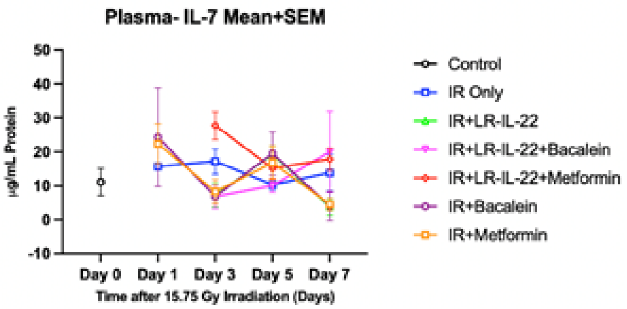
Plasma- IL-7 Mean+SEM
µg/mL Protein
50
40
30
20
10
0
-10
Day 0
Day 1
Day 3
Day 5
Day 7
Time after 15.75 Gy Irradiation (Days)
Control
IR Only
IR+LR-IL-22
IR+LR-IL-22+Bacalein
IR+LR-IL-22+Metformin
IR+Bacalein
IR+Metformin

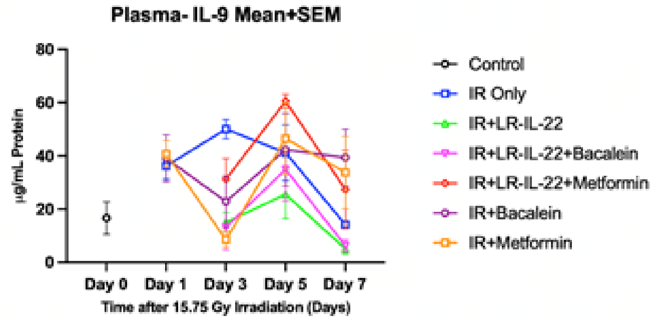
Plasma- IL-9 Mean+SEM
µg/mL Protein
80
60
40
20
0
Day 0
Day 1
Day 3
Day 5
Day 7
Time after 15.75 Gy Irradiation (Days)
Control
IR Only
IR+LR-IL-22
IR+LR-IL-22+Bacalein
IR+LR-IL-22+Metformin
IR+Bacalein
IR+Metformin

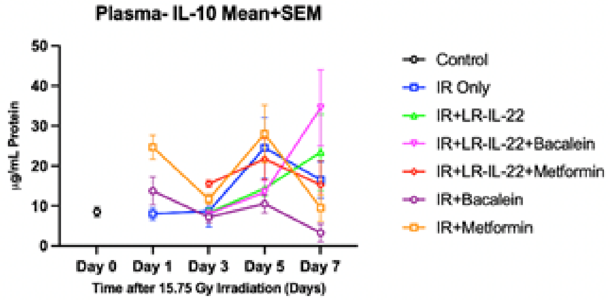
Plasma- IL-10 Mean+SEM
µg/mL Protein
50
40
30
20
10
0
Day 0
Day 1
Day 3
Day 5
Day 7
Time after 15.75 Gy Irradiation (Days)
Control
IR Only
IR+LR-IL-22
IR+LR-IL-22+Bacalein
IR+LR-IL-22+Metformin
IR+Bacalein
IR+Metformin

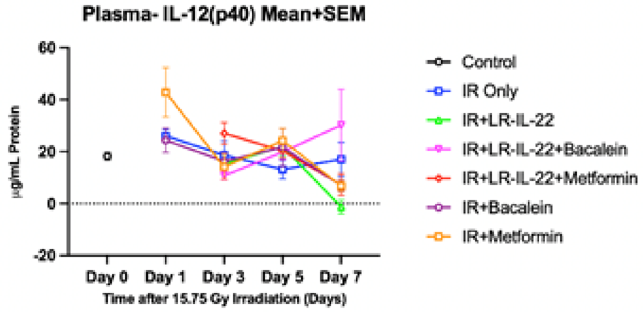
Plasma- IL-12(p40) Mean+SEM
µg/mL Protein
60
40
20
0
-20
Day 0
Day 1
Day 3
Day 5
Day 7
Time after 15.75 Gy Irradiation (Days)
Control
IR Only
IR+LR-IL-22
IR+LR-IL-22+Bacalein
IR+LR-IL-22+Metformin
IR+Bacalein
IR+Metformin

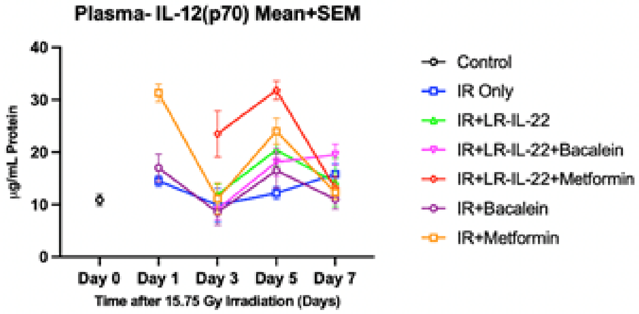
Plasma- IL-12(p70) Mean+SEM
µg/mL Protein
40
30
20
10
0
Day 0
Day 1
Day 3
Day 5
Day 7
Time after 15.75 Gy Irradiation (Days)
Control
IR Only
IR+LR-IL-22
IR+LR-IL-22+Bacalein
IR+LR-IL-22+Metformin
IR+Bacalein
IR+Metformin

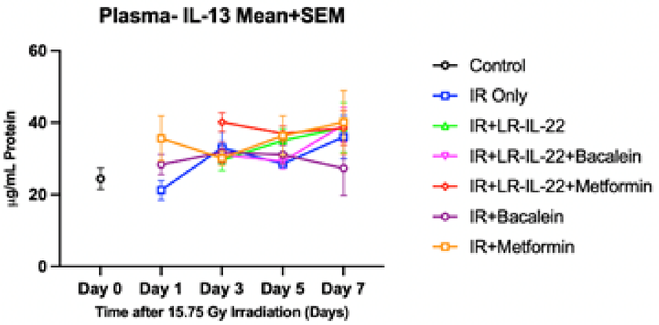
Plasma- IL-13 Mean+SEM
μg/mL Protein
0
20
40
60
Day 0
Day 1
Day 3
Day 5
Day 7
Time after 15.75 Gy Irradiation (Days)
Control
IR Only
IR+LR-IL-22
IR+LR-IL-22+Bacalein
IR+LR-IL-22+Metformin
IR+Bacalein
IR+Metformin

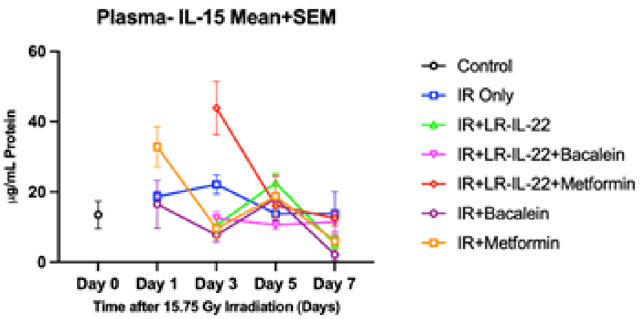
Plasma- IL-15 Mean+SEM
μg/mL Protein
0
20
40
60
Day 0
Day 1
Day 3
Day 5
Day 7
Time after 15.75 Gy Irradiation (Days)
Control
IR Only
IR+LR-IL-22
IR+LR-IL-22+Bacalein
IR+LR-IL-22+Metformin
IR+Bacalein
IR+Metformin

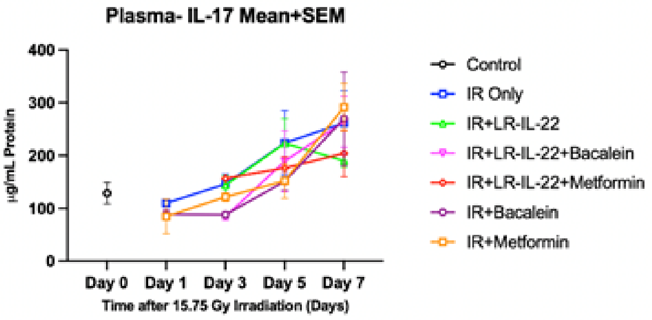
Plasma- IL-17 Mean+SEM
μg/mL Protein
0
100
200
300
400
Day 0
Day 1
Day 3
Day 5
Day 7
Time after 15.75 Gy Irradiation (Days)
Control
IR Only
IR+LR-IL-22
IR+LR-IL-22+Bacalein
IR+LR-IL-22+Metformin
IR+Bacalein
IR+Metformin

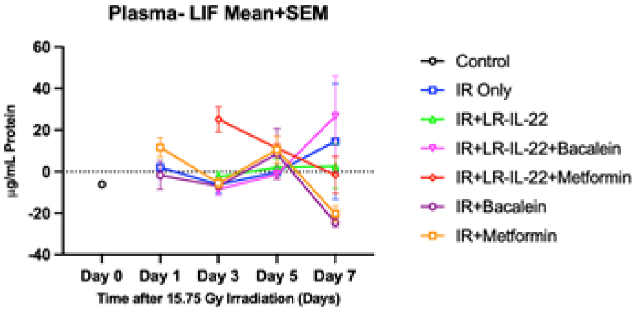
Plasma- LIF Mean+SEM
μg/mL Protein
-40
-20
0
20
40
60
Day 0
Day 1
Day 3
Day 5
Day 7
Time after 15.75 Gy Irradiation (Days)
Control
IR Only
IR+LR-IL-22
IR+LR-IL-22+Bacalein
IR+LR-IL-22+Metformin
IR+Bacalein
IR+Metformin

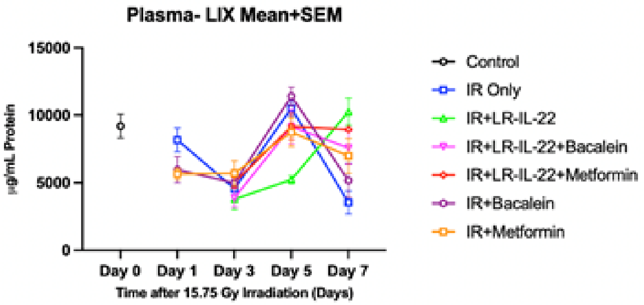
Plasma- LIX Mean+SEM
μg/mL Protein
0
5000
10000
15000
Day 0
Day 1
Day 3
Day 5
Day 7
Time after 15.75 Gy Irradiation (Days)
Control
IR Only
IR+LR-IL-22
IR+LR-IL-22+Bacalein
IR+LR-IL-22+Metformin
IR+Bacalein
IR+Metformin

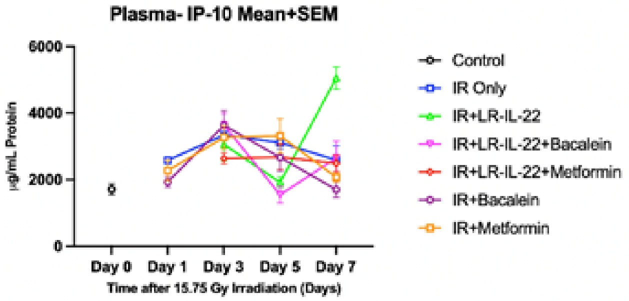
Plasma- IP-10 Mean+SEM
μg/mL Protein
0
2000
4000
6000
Day 0
Day 1
Day 3
Day 5
Day 7
Time after 15.75 Gy Irradiation (Days)
Control
IR Only
IR+LR-IL-22
IR+LR-IL-22+Bacalein
IR+LR-IL-22+Metformin
IR+Bacalein
IR+Metformin

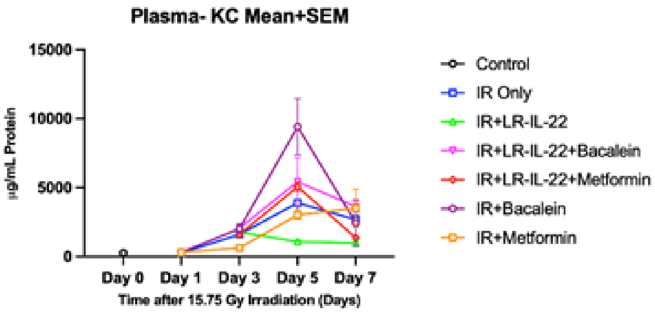
Plasma- KC Mean+SEM
μg/mL Protein
0
5000
10000
15000
Day 0
Day 1
Day 3
Day 5
Day 7
Time after 15.75 Gy Irradiation (Days)
Control
IR Only
IR+LR-IL-22
IR+LR-IL-22+Bacalein
IR+LR-IL-22+Metformin
IR+Bacalein
IR+Metformin

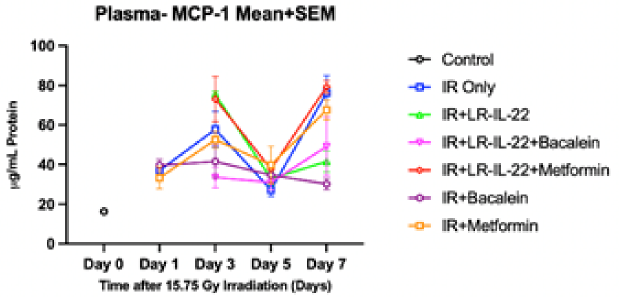
Plasma- MCP-1 Mean+SEM
μg/mL Protein
0
20
40
60
80
100
Day 0
Day 1
Day 3
Day 5
Day 7
Time after 15.75 Gy Irradiation (Days)
Control
IR Only
IR+LR-IL-22
IR+LR-IL-22+Bacalein
IR+LR-IL-22+Metformin
IR+Bacalein
IR+Metformin

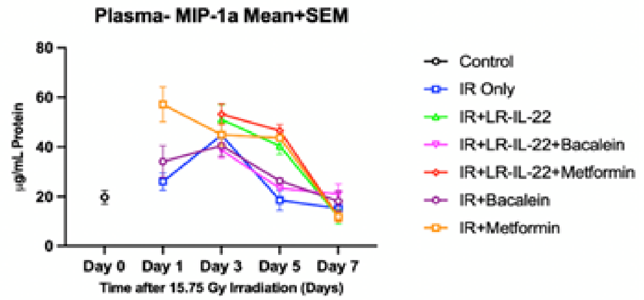
Plasma- MIP-1a Mean+SEM
µg/mL Protein
0
20
40
60
80
Day 0
Day 1
Day 3
Day 5
Day 7
Time after 15.75 Gy Irradiation (Days)
Control
IR Only
IR+LR-IL-22
IR+LR-IL-22+Bacalein
IR+LR-IL-22+Metformin
IR+Bacalein
IR+Metformin

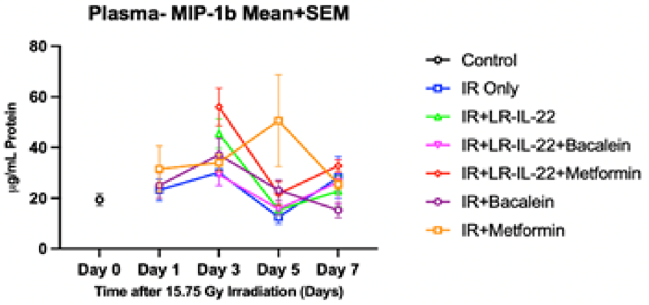
Plasma- MIP-1b Mean+SEM
µg/mL Protein
0
20
40
60
80
Day 0
Day 1
Day 3
Day 5
Day 7
Time after 15.75 Gy Irradiation (Days)
Control
IR Only
IR+LR-IL-22
IR+LR-IL-22+Bacalein
IR+LR-IL-22+Metformin
IR+Bacalein
IR+Metformin

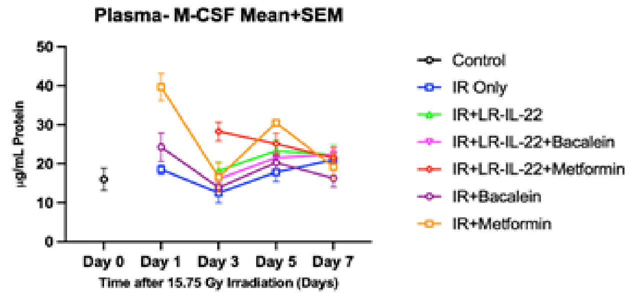
Plasma- M-CSF Mean+SEM
µg/mL Protein
0
10
20
30
40
50
Day 0
Day 1
Day 3
Day 5
Day 7
Time after 15.75 Gy Irradiation (Days)
Control
IR Only
IR+LR-IL-22
IR+LR-IL-22+Bacalein
IR+LR-IL-22+Metformin
IR+Bacalein
IR+Metformin

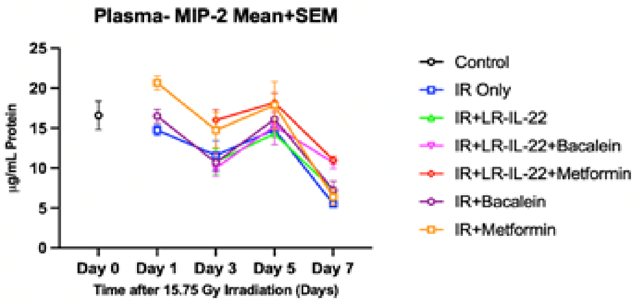
Plasma- MIP-2 Mean+SEM
µg/mL Protein
0
5
10
15
20
25
Day 0
Day 1
Day 3
Day 5
Day 7
Time after 15.75 Gy Irradiation (Days)
Control
IR Only
IR+LR-IL-22
IR+LR-IL-22+Bacalein
IR+LR-IL-22+Metformin
IR+Bacalein
IR+Metformin

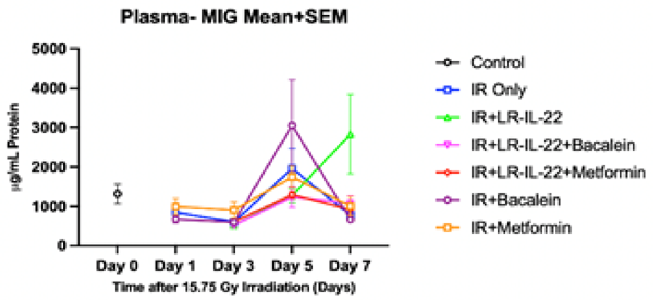
Plasma- MIG Mean+SEM
µg/mL Protein
0
1000
2000
3000
4000
5000
Day 0
Day 1
Day 3
Day 5
Day 7
Time after 15.75 Gy Irradiation (Days)
Control
IR Only
IR+LR-IL-22
IR+LR-IL-22+Bacalein
IR+LR-IL-22+Metformin
IR+Bacalein
IR+Metformin

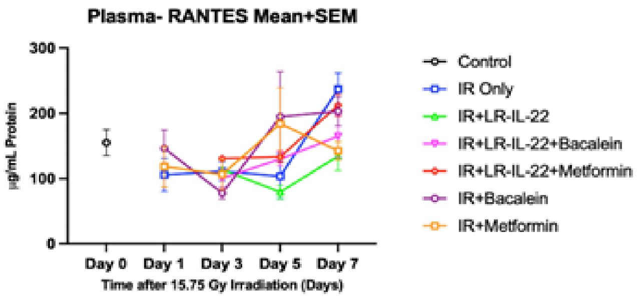
Plasma- RANTES Mean+SEM
µg/mL Protein
0
100
200
300
Day 0
Day 1
Day 3
Day 5
Day 7
Time after 15.75 Gy Irradiation (Days)
Control
IR Only
IR+LR-IL-22
IR+LR-IL-22+Bacalein
IR+LR-IL-22+Metformin
IR+Bacalein
IR+Metformin

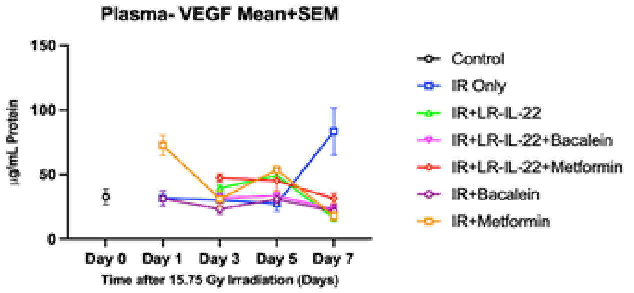
Plasma- VEGF Mean+SEM
µg/mL Protein
0
50
100
150
Day 0
Day 1
Day 3
Day 5
Day 7
Time after 15.75 Gy Irradiation (Days)
Control
IR Only
IR+LR-IL-22
IR+LR-IL-22+Bacalein
IR+LR-IL-22+Metformin
IR+Bacalein
IR+Metformin

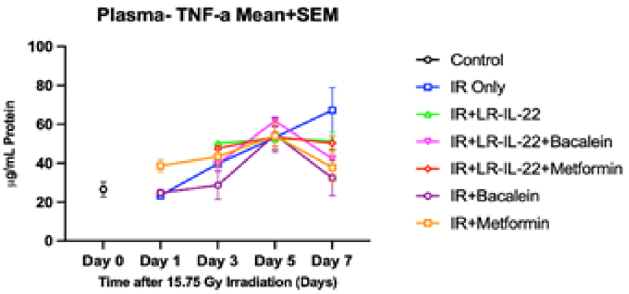
Plasma- TNF-a Mean+SEM
µg/mL Protein
0
20
40
60
80
100
Day 0
Day 1
Day 3
Day 5
Day 7
Time after 15.75 Gy Irradiation (Days)
Control
IR Only
IR+LR-IL-22
IR+LR-IL-22+Bacalein
IR+LR-IL-22+Metformin
IR+Bacalein
IR+Metformin

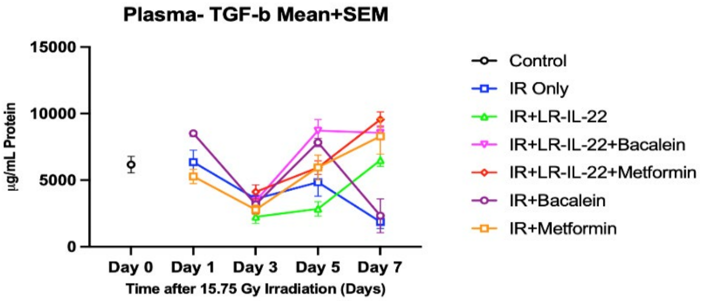
Plasma- TGF-b Mean+SEM
15000
10000
5000
0
µg/mL Protein
Day 0
Day 1
Day 3
Day 5
Day 7
Time after 15.75 Gy Irradiation (Days)
Control
IR Only
IR+LR-IL-22
IR+LR-IL-22+Bacalein
IR+LR-IL-22+Metformin
IR+Bacalein
IR+Metformin
